# Enhancing the Predictive Power of Macrocyclic Drug Permeability by Knowledge Distillation from Analogous Pretraining Data

**DOI:** 10.1021/acs.jmedchem.5c02620

**Published:** 2025-12-20

**Authors:** Yu Zhang, Olli T. Pentikäinen

**Affiliations:** † Institute of Biomedicine, Integrative Physiology and Pharmacy, 8058 University of Turku, FI-20014 Turku, Finland; ‡ InFLAMES Research Flagship Center, University of Turku, FI-20014 Turku, Finland

## Abstract

Macrocyclic drugs offer powerful opportunities for modulating protein–protein interactions, yet their development is limited by poor and unpredictable membrane permeability. Experimental testing is slow, and 3D modeling of macrocycles is computationally demanding due to their large conformational space. To address this, we present Multi_DDPP, a deep learning (DL) model that predicts macrocycle permeability directly from 2D structures. Multi_DDPP employs knowledge distillation to leverage permeability data from multiple cell lines, improving generalizability, and uses a task-specific swing-range strategy to reduce label noise. By integrating diverse molecular representations, including physicochemical descriptors, fingerprints, molecular graphs, and hybrid features, the model outperforms existing ML and DL approaches. Node masking highlights the substructures that contribute most to permeability, and regression extensions incorporating physiological parameters further refine these predictions. Early 2D-based permeability prediction with Multi_DDPP avoids the costly generation of 3D conformers and enables the efficient prioritization of macrocycles with favorable pharmacokinetic potential.

## Graphical abstract

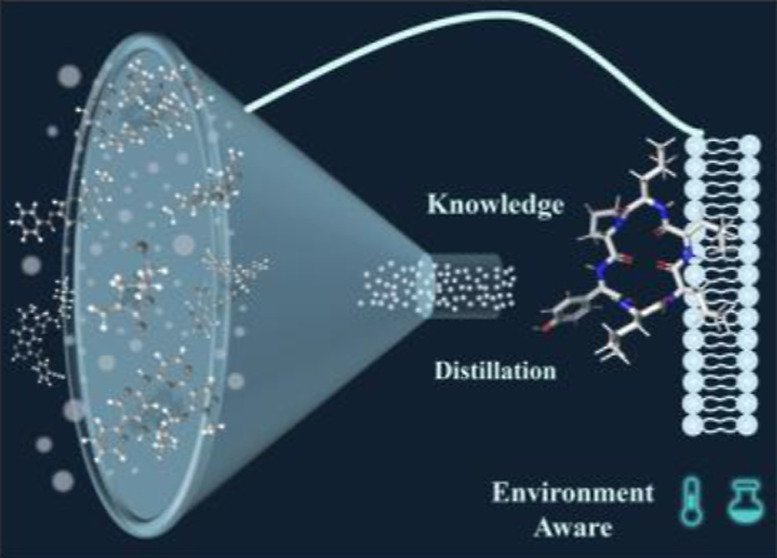

## Introduction

The searchable chemical universe has expanded dramatically, with libraries such as ZINC and Enamine REAL enumerating billions of synthetically accessible small molecules, making exhaustive experimental profiling infeasible.^1–4^ For macrocycles, the challenge is even greater: their conformational flexibility, stereochemical complexity, and beyond-Rule-of-Five properties create a vastly larger and more intricate design space. This scale explosion underscores the need for fast and accurate in silico filters to prioritize candidates before synthesis.

Advances in computational resources enable the development of more technologies for processing complex patterns, such as virtual high-throughput screening^5,6^ and various artificial intelligence application scenarios and methods,^7^ particularly in drug discovery.^8–10^ Deep learning (DL) has emerged as a powerful approach for modeling complex structure–property relationships, leveraging graph-based and descriptor representations to outperform traditional QSAR methods in ADME prediction.^11–13^ However, DL models in drug discovery often face label scarcity, heterogeneous assay conditions, and label noise, which degrade predictive reliability.^14–16^ To address these issues, some approaches reduce the cost of expert annotation, while others rely on substantial compounds without labels^17–20^ or generate pseudo-labels^21^ to assist pretraining models. For instance, unsupervised learning models, such as those pretraining unlabeled molecules, extract more molecular representation compared with traditional descriptors, like fingerprint-based features,^22^ pharmacophore-based features,^23^ or semi-supervised learning models that use pseudo-labels generated by transductive label propagation based on the manifold assumption, in which similar samples have the same label. Unfortunately, for task-specific models in drug discovery, this fine-grained classification can be influenced due to the confirmation bias,^24–27^ and slight structure differences also lead to opposite results. Sufficiently utilizing similar labels based on chemical knowledge is effective in enhancing DL models.

Macrocycles are increasingly recognized as privileged scaffolds for modulating protein–protein interactions and other challenging targets; however, their therapeutic potential is often limited by poor membrane permeability, a key determinant of oral bioavailability.^14,15^ The basic knowledge of optimizing permeability when designing small molecules can also be extended into macrocycles, such as enhancing intramolecular hydrogen bonding that contributes to conformational shifts, facilitating membrane permeability.^28–30^ Considering different situations of permeation of diverse compounds can offer extensive opportunities to comprehensively obtain potential relationships between structures and permeability. Meanwhile, AI-driven macrocycle design—including recent frameworks capable of generating millions of structured macrocycles with experimentally validated permeability and oral bioavailability—further underscores the need for scalable, accurate permeability prediction.^31,32^ In parallel, high-throughput synthetic strategies now enable the rapid generation of thousands of structurally diverse macrocycles using modular chemistries and encoded libraries,^33^ making computational prescreening essential to focus experimental resources on the most promising candidates. However, the current approaches for predicting macrocycle permeability have some shortcomings. Established physicochemical and theoretical models^30,34,35^ are computationally demanding and scale poorly, limiting their applicability to high-throughput screening. Recent DL methods, including Multi_CycGT^36^ and CycPeptMP,^37^ are trained on single-assay data sets and therefore fail to capture broader permeability-relevant features. Other efforts, such as the assay-based classification framework,^38^ treat data sets independently, preventing the transfer of useful information across related assays and constrain overall predictive performance.

Here, we introduce Multi_DDPP, a DL framework that combines knowledge distillation with multi-representation learning to leverage large, noisy data sets while improving performance on curated macrocycle-specific data. By integrating complementary information from multiple assays measuring the same property, Multi_DDPP enhances the data efficiency and predictive accuracy. Multi_DDPP mitigates label noise, integrates graph and descriptor features, and provides substructure-level interpretability, enabling early stage permeability screening to accelerate macrocycle drug design.

## Results

Balancing potency and permeability remains a significant challenge in the discovery of macrocyclic drugs. Poor membrane permeability often limits oral absorption and contributes to attrition during preclinical development. Although the conformational rigidity and structural complexity of macrocycles can enhance target binding, these same features complicate the reliable assessment of the permeability. As a result, accurate permeability prediction is essential throughout the macrocycle optimization. Recent advances in machine learning and DL have enabled more efficient and cost-effective strategies for estimating permeability and prioritizing compounds with favorable developability profiles.

### Framework of Multi_DDPP

To establish a foundation for permeability prediction in macrocyclic drug development, we first trained a baseline model on a large, low-fidelity data set containing 23,086 molecules, including both macrocycles and small molecules. This broad data set was selected to maximize chemical diversity and the number of labeled examples. Because permeability measurements across assays are heterogeneous, we introduced a task-specific swing-range formulation to mitigate label noise while retaining sufficient data for DL. To capture complementary information relevant to membrane transport, we incorporated permeability data from multiple assay formats—including Caco-2, MDCK, RRCK, and PAMPA—which collectively broadened the representation of passive diffusion and transporter effects. After pretraining on the large, multi-assay data set, we transferred dark information into the specific task (single assay and macrocycles). The resulting framework, Multi_DDPP (Figure 1), integrates graph-based and descriptor-based molecular representations while leveraging latent knowledge distilled from the broader data set. We benchmarked Multi_DDPP against multiple feature types—descriptors, fingerprints, graphs, and hybrid combinations—across a range of machine learning and DL models.

**Figure 1:**
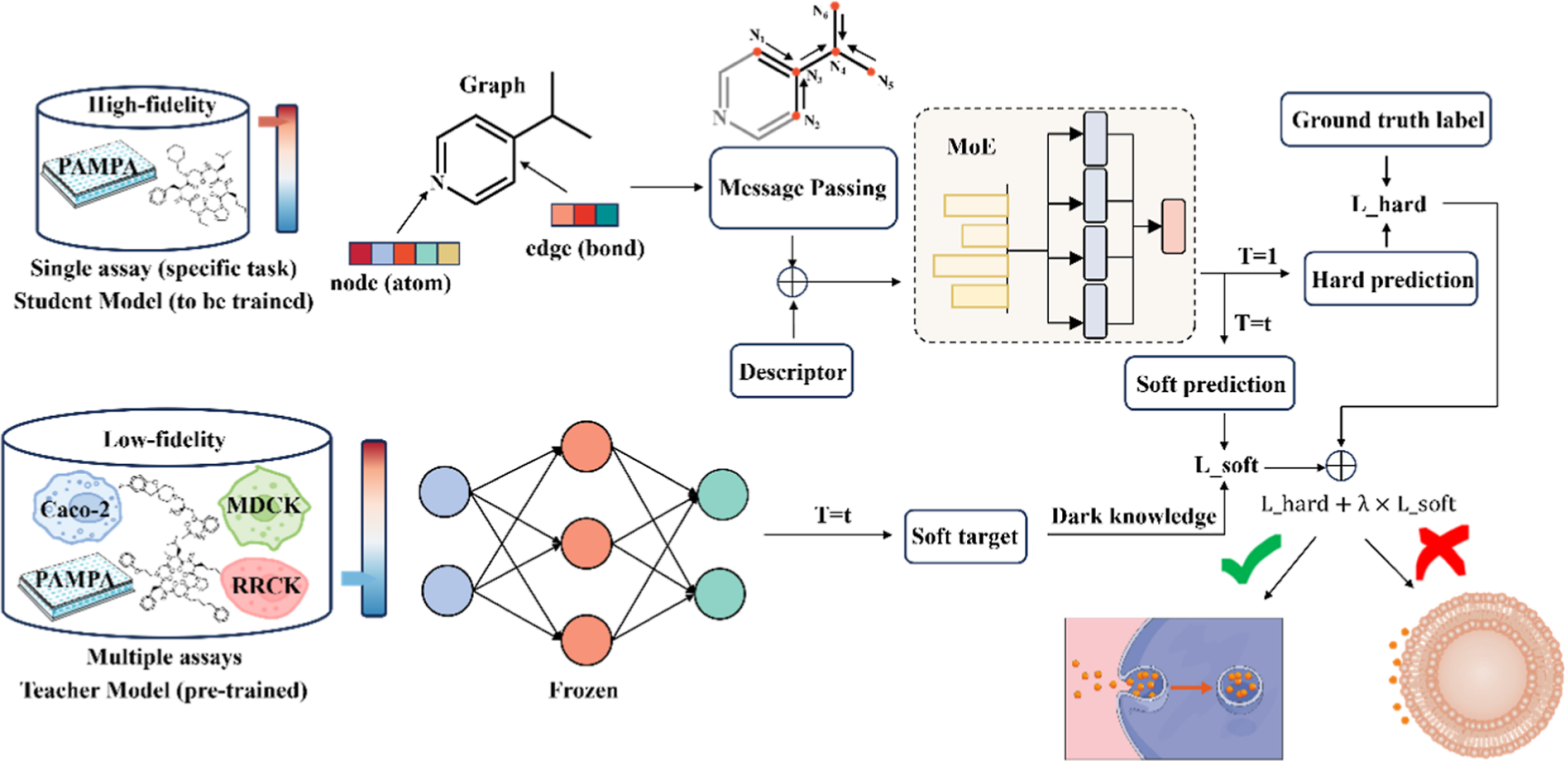
Overview of the Multi_DDPP model for prediction of macrocycles’ permeability.

### Fidelity of the Data Set

Noise in labeling can significantly bias models and pose a significant obstacle to the subsequent study. Often, thresholds are applied to segment data without considering the quality of the data. Here, we collected permeability data for 227 macrocycles tested by different research groups. Different experimental environments and other factors cause deviation. We defined a swing range that minimizes the impact of noise tags to the greatest extent possible. Approximately 77.5% of the data show a difference in −log *P* of less than 0.5, while only 22.5% exceed this threshold. Notably, within this data, around 31.7% of macrocycles have conflicting PAMPA permeability data, one measurement below 1 × 10^–6^ cm/s and another above, affecting classification as permeable or not (Figure 2A). We aimed to reduce noise while retaining as much data as possible. Accordingly, we set the range of −log *P* between 5.5 and 6.5 as the swing range and excluded data within this range to avoid misleading labels. Furthermore, although many macrocycles fall beyond Rule of Five (Ro5), which is commonly used to evaluate pharmacokinetic properties, more than half of them can still be permeable (Figure S1). This indicates a broad chemical space to explore for potential drug candidates.

**Figure 2:**
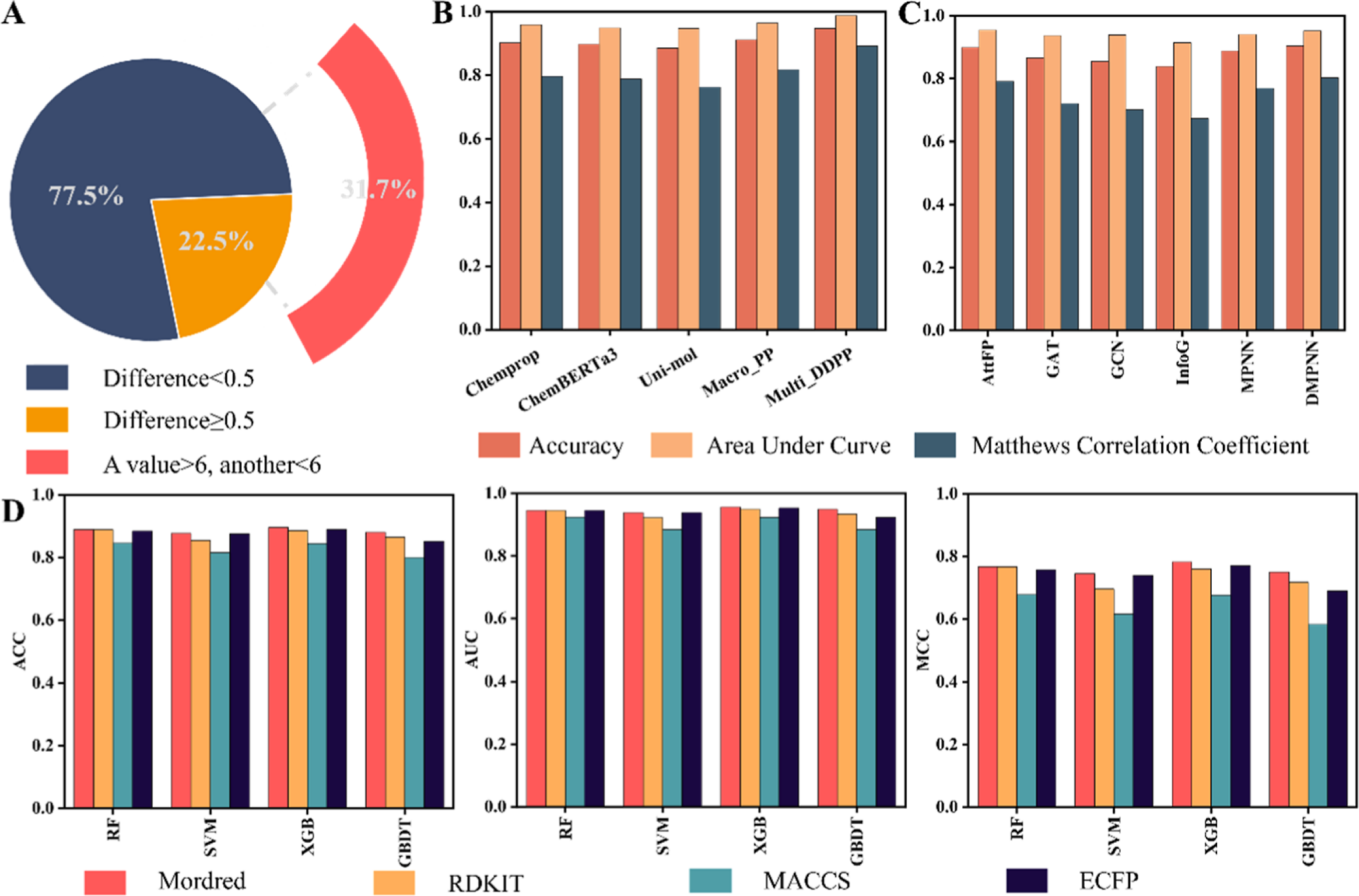
High-fidelity data set and performance evaluation of Multi_DDPP compared with baseline models. (A) A total of 227 pairs of PAMPA permeability values, where each pair represents independent experimental conditions. A swing range was defined based on these data to maximize retention and reduce the impact of noisy labels. (B) The ACC, area under the curve (AUC), and MMC performances of models combining molecular graphs with descriptors: Chemprop, Macro_PP, Multi_DDPP, and two pretrained models: ChemBERTa-3 and Uni-mol. (C) The ACC, AUC, and MMC performances of graph-based models (AttentiveFP, GAT, GCN, InfoGraph, MPNN, DMPNN) are shown. (D) The ACC, AUC, and MMC performances of traditional machine learning models (RF, SVM, XGB, GBDT) using Mordred, Rdkit, MACCS, and ECFP descriptors.

### Performance of Multi_DDPP

To facilitate the Multi_DDPP validation, we compared the performance of our model with four traditional machine learning methods (RF, SVM, XGB, and GBDT)—as well as five graph-based DL models: AttentiveFP, GAT, GCN, InfoGraph, MPNN, and DMPNN. In addition, we included Chemprop, which combines molecular graph features with selected descriptors (Table S1). Furthermore, we also trained the data using other pretrained models, including ChemBERTa-3 and Uni-mol. To ensure comprehensive usage of features in model development, we used various molecular representations, including traditional features, molecular descriptors (Rdkit2D and Mordred), molecular fingerprints (ECFP and MACCS), two-dimensional representations, molecular graphs, and hybrid representations that combine descriptors with molecular graphs. We also assessed the impact of knowledge distillation by comparing our model’s performance with and without it, demonstrating the advantage of our approach.

To demonstrate the robustness of our model, we performed 10-fold cross-validation using SMILES string-based delineation criteria to avoid data leakage. The evaluation results indicate that our model achieves optimal performance across multiple models and molecular representations. In the model without knowledge distillation, referred to as Macro_PP, we employed a mixture of expert (MoE) framework to address more complicated and extensive prediction tasks. Notably, Macro_PP outperformed all baseline models. For instance, the accuracy of Macro_PP (ACC = 0.912) is higher than that of traditional machine learning models based on different descriptors and fingerprints: RF_R, RF_M, RF_EF, RF_MF, SVM_R, SVM _M, SVM _EF, SVM _MF, XGB_R, XGB _M, XGB _EF, XGB _MF, GBDT_R, GBDT _M, GBDT _EF, and GBDT _MF (Table S2), and the improvement in ACC ranged from 1.7% to 11.3% compared to these methods (Figure 2B,D). We further evaluated Matthews correlation coefficient (MCC) (0.818) and AUC (0.964) values to demonstrate the robustness of Macro_PP, which straightforwardly suggests the stability of the model prediction. Macro_PP is superior to all traditional machine models, the elevated range of AUC values is from 0.8% to 8% (Figure 2B,D), and the elevated range of MCC values is from 3.6% to 23.6% (Figure 2B,D). Furthermore, we compared Macro_PP with graph-based DL models (AttentiveFP, GAT, GCN, InfoGraph, MPNN, and DMPNN) using the same metrics (Table S3). Macro_PP again showed superior performance with improvements ranging from 1.3% to 8.3% in ACC, 0.9% to 5% in AUC, and 2.7% to 14.5% in MCC (Figure 2B,C). To eliminate the effect of molecular representation, we compared Macro_PP to Chemprop, which also combines descriptors and a molecular graph. Macro_PP still showed improvements: 1% in ACC, 0.5% in AUC, and 2.1% in MCC (Figure 2B).

We further compared Multi_DDPP to all baseline models including Macro_PP. By applying knowledge distillation from a large data set with a broad chemical space, Multi _DDPP was able to incorporate not only the traditional molecular representations and molecular graphs but also latent knowledge extracted from the large data set. This additional information helped the model to achieve more accurate predictions. For instance, compared with machine learning models, Multi_DDPP obtains improvements in ACC (ACC = 0.948) from 5.3% to 14.9%, AUC (AUC = 0.988) from 3.2% to 10.4%, and MCC (AUC = 0.892) from 11% to 31%, individually (Figure 2B,D). Multi_DDPP also outperformed models based on other molecular representations. Due to shared skeletons among different molecules, which can contribute to light data leakage, we split data based on Murcko scaffolds to mitigate the effects of shared scaffolds. There is a slight decline in performance for Macro_PP (ACC = 0.890, AUC = 0.939, MCC = 0.765) and Multi_DDPP (ACC = 0.933, AUC = 0.978, MCC = 0.857); however, they remain outstanding and have stable predictive ability (Table S4). Compared to other models, Multi_DDPP substantially outperforms them, indicating its effectiveness in predicting the permeability of macrocyclic molecules.

In summary, compared with all baseline models, the comprehensive evaluation demonstrates that Multi_DDPP is a highly effective method to predict permeability. By distilling latent information from a large data set, it can transfer valuable knowledge to more targeted prediction tasks. This highlights its potential as a powerful approach for leveraging available labeled data to support specific modeling challenges.

### Performance of Multi_DDPP across Noise Levels

To assess the robustness of Multi_DDPP, we evaluated its performance on data sets constructed using different swing values (0.2, 0.4, 0.6, 0.8), which modulate data set size and label noise. Across all noise levels, the proportion of impermeable and permeable compounds remained relatively consistent at approximately 4:6 (Figure S2). For clarity, each data set was further divided by macrocycle ring size (12–15, 16–18, and >18 atoms), and the corresponding data counts are shown in Figure S3.

Across all four noise settings—from high noise (0.2) to very low noise (0.8)—Multi_DDPP consistently achieved the best overall predictive performance. In the very low-noise data set (swing = 0.8), Multi_DDPP improved ACC (0.981) by 3.9–12.9%, AUC (0.998) by 1.7–12.5%, MCC (0.961) by 8.0–25.2%, and PR-AUC (0.998) by 1.5–13.2% relative to baseline models (Tables 1 and S10). Macro_PP ranked second across all metrics.

**Table 1:** Baseline DL Models, Macro_PP, and Multi_DDPP (Swing Value = 0.8)

	ACC	AUC	MCC	PR-AUC
AttentiveFP	0.942 ± 0.009	0.980 ± 0.007	0.881 ± 0.018	0.981 ± 0.010
GAT	0.909 ± 0.023	0.967 ± 0.012	0.816 ± 0.042	0.973 ± 0.010
GCN	0.915 ± 0.018	0.972 ± 0.013	0.829 ± 0.038	0.977 ± 0.011
InfoGraph	0.871 ± 0.036	0.873 ± 0.033	0.742 ± 0.062	0.866 ± 0.037
MPNN	0.930 ± 0.016	0.970 ± 0.009	0.856 ± 0.036	0.972 ± 0.009
Chemprop	0.935 ± 0.007	0.980 ± 0.002	0.866 ± 0.017	0.983 ± 0.006
DMPNN	0.939 ± 0.009	0.971 ± 0.010	0.876 ± 0.014	0.969 ± 0.013
ChemBERTa-3	0.936 ± 0.013	0.981 ± 0.004	0.871 ± 0.027	0.981 ± 0.004
Uni-mol	0.925 ± 0.014	0.939 ± 0.012	0.847 ± 0.027	0.980 ± 0.004
Macro_PP	0.955 ± 0.010	0.984 ± 0.006	0.907 ± 0.022	0.985 ± 0.007
Multi_DDPP	0.981 ± 0.010	0.998 ± 0.002	0.961 ± 0.020	0.998 ± 0.001

Similarly, in the low-noise data set (swing = 0.6), Multi_DDPP achieved ACC = 0.964, AUC = 0.992, MCC = 0.925, and PR-AUC = 0.995, corresponding to improvements of 3.3–15.1%, 1.5–15.5%, 6.8–32.1%, and 1.3–15.1%, respectively (Tables S6 and S9). Macro_PP again provided the second-best performance.

In the high-noise data set (swing = 0.2), Multi_DDPP remained robust, with ACC = 0.915, AUC = 0.972, MCC = 0.822, and PR-AUC = 0.981—representing improvements of 3.8–14.4%, 3.7–18.3%, 7.6–30.6%, and 2.9–28.4%, respectively (Tables 2 and S7). Similar trends were observed at swing = 0.4 (ACC = 0.938, AUC = 0.983, MCC = 0.872, PR-AUC = 0.988), with improvements of 3.9–14.9%, 1.5–18.3%, 6.8–31.6%, and 1.3–18.0%, respectively (Tables S5 and S8).

**Table 2:** Baseline DL Models, Macro_PP, and Multi_DDPP (Swing Value = 0.2)

	ACC	AUC	MCC	PR-AUC
AttentiveFP	0.868 ± 0.012	0.927 ± 0.011	0.724 ± 0.027	0.941 ± 0.012
GAT	0.809 ± 0.029	0.886 ± 0.026	0.599 ± 0.067	0.913 ± 0.017
GCN	0.839 ± 0.014	0.915 ± 0.007	0.663 ± 0.032	0.936 ± 0.006
InfoGraph	0.790 ± 0.023	0.789 ± 0.019	0.572 ± 0.041	0.797 ± 0.018
MPNN	0.859 ± 0.013	0.923 ± 0.008	0.705 ± 0.029	0.938 ± 0.007
Chemprop	0.871 ± 0.010	0.935 ± 0.008	0.732 ± 0.021	0.952 ± 0.007
DMPNN	0.877 ± 0.013	0.933 ± 0.012	0.746 ± 0.026	0.943 ± 0.014
ChemBERTa-3	0.857 ± 0.013	0.915 ± 0.008	0.700 ± 0.026	0.930 ± 0.009
Uni-mol	0.852 ± 0.011	0.919 ± 0.006	0.692 ± 0.022	0.939 ± 0.006
Macro_PP	0.879 ± 0.015	0.942 ± 0.008	0.746 ± 0.033	0.954 ± 0.007
Multi_DDPP	0.915 ± 0.010	0.972 ± 0.009	0.822 ± 0.027	0.981 ± 0.007

Overall, these evaluations demonstrate that Multi_DDPP consistently provides state-of-the-art predictive performance across data sets with varying noise levels, highlighting its robustness and suitability for real-world macrocycle permeability prediction.

To examine how ring size influences predictive performance, we partitioned the data set (swing = 0.5) into three categories: small rings (12–15 atoms), medium rings (16–18 atoms), and large rings (>18 atoms). This allowed us to compare model behavior across distinct structural regimes while maintaining adequate sample sizes.

For both Multi_DDPP and Macro_PP, performance improved in the small and medium ring subsets relative to the full data set, indicating strong applicability in these chemically tractable regions. In contrast, performance declined for large-ring macrocycles (>18 atoms) (Figure S4). This reduction is consistent with the increased conformational complexity and greater physicochemical variability of large rings, which likely require more detailed structural information to model accurately.

### Evaluation of the Effects of the Large Data Set

To further explore the effects of the large data set on model performance, we used three different strategies to split the high-fidelity data set. In each iteration, we added 10% of high-fidelity data into the large data set and evaluated model performance using consistent metrics. This approach allowed us to assess whether the quality of the large data set influences knowledge transfer. We used three splitting strategies: (1) molecular fingerprint-based split, (2) scaffold-based split, and (3) random split (Table S11).

For the fingerprint-based split, we computed ECFP fingerprints of the high-fidelity data set and reduced them into two dimensions using t-SNE (Figure 3A). We used the elbow method and silhouette score to confirm the best cluster number (Figure S5). For the scaffold-based split, we computed Tanimoto similarity between scaffold pairs (Figure 3B) and clustered the data set accordingly. Furthermore, when evaluating the models, we also compared the number of repetitive data points in every two different strategies, avoiding excessive duplicates that pose a threat to evaluating the effect of the large data set. It shows that the repetition rate of added high-fidelity data increases from an average of 39% to 78% when adding 40% data to 80% data (Figure 3C). Furthermore, as more high-fidelity data was incorporated, the model got continuous improvement with a decreasing proportion of noise labels (Figure S6).

**Figure 3:**
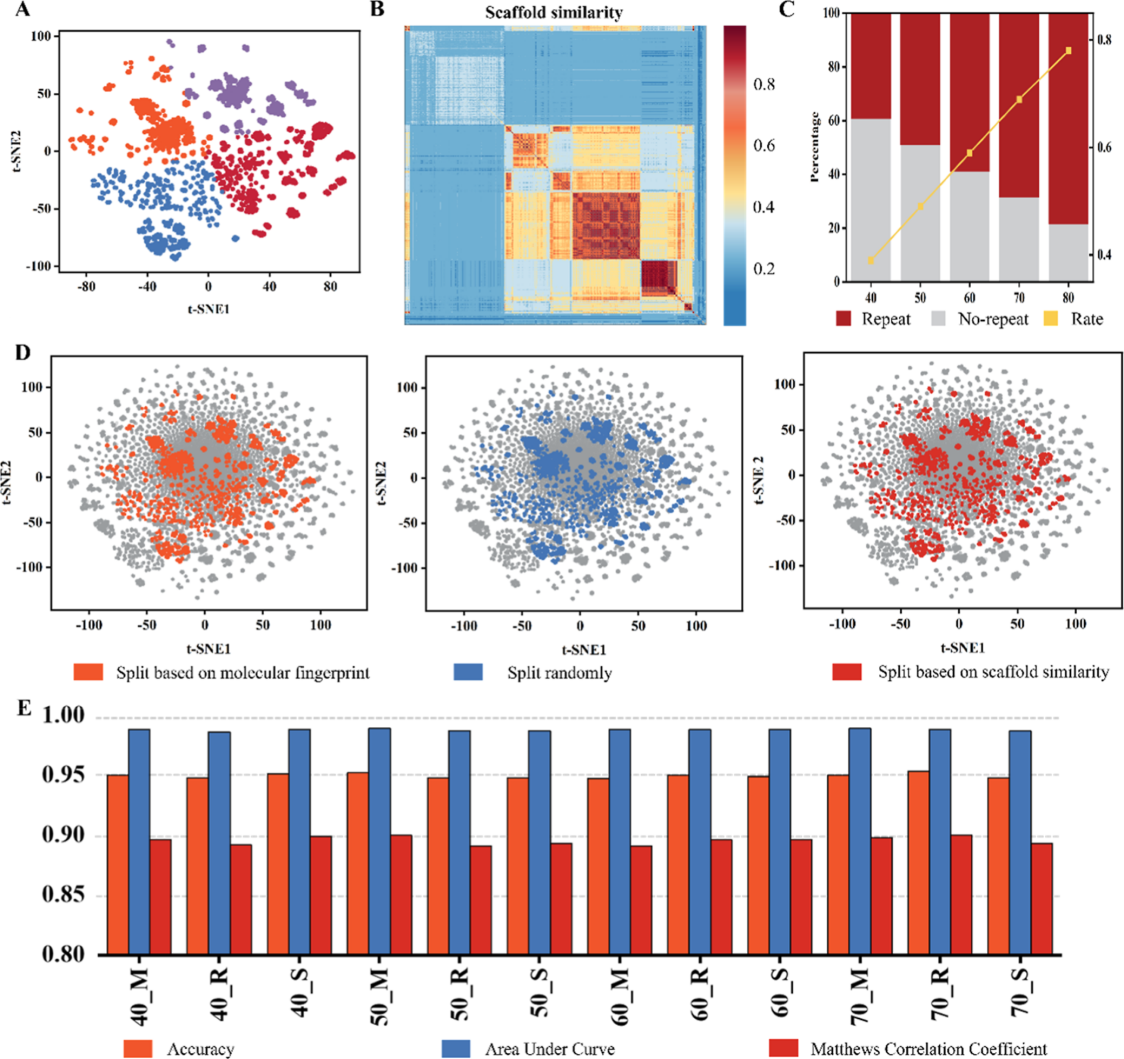
Evaluation of the effects of the large data set. (A) The t-SNE visualization is used to present four clusters of high-fidelity data based on the molecular fingerprint properties. (B) Computed Tanimoto similarity of every two scaffolds of macrocycles for clustering the high-fidelity data set. (C) The proportion of repeated molecules between each two different strategies increases when more high-fidelity data is added to the large data set. (D) The t-SNE visualizations of different proportions of added high-fidelity data based on different split strategies in the large data set, in which the model achieves the optimal result, are shown. (E) The ACC, ACU, and MCC performances of Multi_DDPP when adding different proportions of high-fidelity data based on different split strategies.

We evaluated Multi_DDPP across different large data sets, comparing their performances using consistent metrics. Multi_DDPP achieved notable improvements. Regarding the different splitting strategies, the model achieved optimal effectiveness at varying rates of added high-fidelity data: 50% for the fingerprint split, 40% for the scaffold split, and 70% for the random split (Figure 3D). Additional proportions of added high-fidelity data are also shown (Figure S7). The best performance for fingerprint split was ACC = 0.952, AUC = 0.989, and MCC = 0.900; for scaffold split, ACC = 0.951, AUC = 0.988, and MCC = 0.899; and for random split, ACC = 0.953, AUC = 0.989, and MCC = 0.900 (Figure 3E). Evaluations of other different proportions of added high-fidelity data models are shown in Figure S8 and Table S12, which shows that some of them achieve minor enhancements. This indicates that Multi_DDPP can extract more valuable information from larger data sets.

### Feature Importance

To explore which features significantly impact the permeability of macrocycles, we applied a masking strategy to the node features (Figure 4A). By comparing the differences in loss with and without a mask on each node feature, we quantified the importance of node features based on the △loss of node features. The strength of H-bonds that molecules make with water significantly influences whether the molecules can transfer from water to nonpolar environments easily, which reflects the oral availability of drugs. Elimination of nonessential hydrogen-bond donors is a well-established strategy for enhancing oral bioavailability, particularly in the design of macrocyclic drugs.^39^ Consistent with this, our model identifies hydrogen-bond donors as strong determinants of permeability (Figure 4B). Additionally, lone pairs have emerged as another influential feature. Due to the complexity of drug molecules, and considering that approximately 63% of FDA-approved drugs between 2015 and 2020 are chiral,^40^ lone pairs contribute to asymmetric charge distributions and dipole moments. These properties influence molecular polarity and, consequently, membrane permeability. Similarly, rigidity impacts oral availability. Almost all orally available peptides are cyclic,^41^ and cyclization is an effective strategy to reduce the flexibility of molecules. The node feature indicating whether the atom is part of a large ring reflects the rigidity of molecules, which, in turn, impacts permeability. We further evaluated the contribution of the combined descriptors. Compared with graph-only models, models integrating global descriptors showed modest performance gains (Macro_PP: ACC = 0.904, AUC = 0.963, MCC = 0.801, PR-AUC = 0.968, Multi_DDPP: ACC = 0.937, AUC = 0.984, MCC = 0.869, PR-AUC = 0.988) (Table S13), indicating that global molecular features complement graph-based representations.

**Figure 4:**
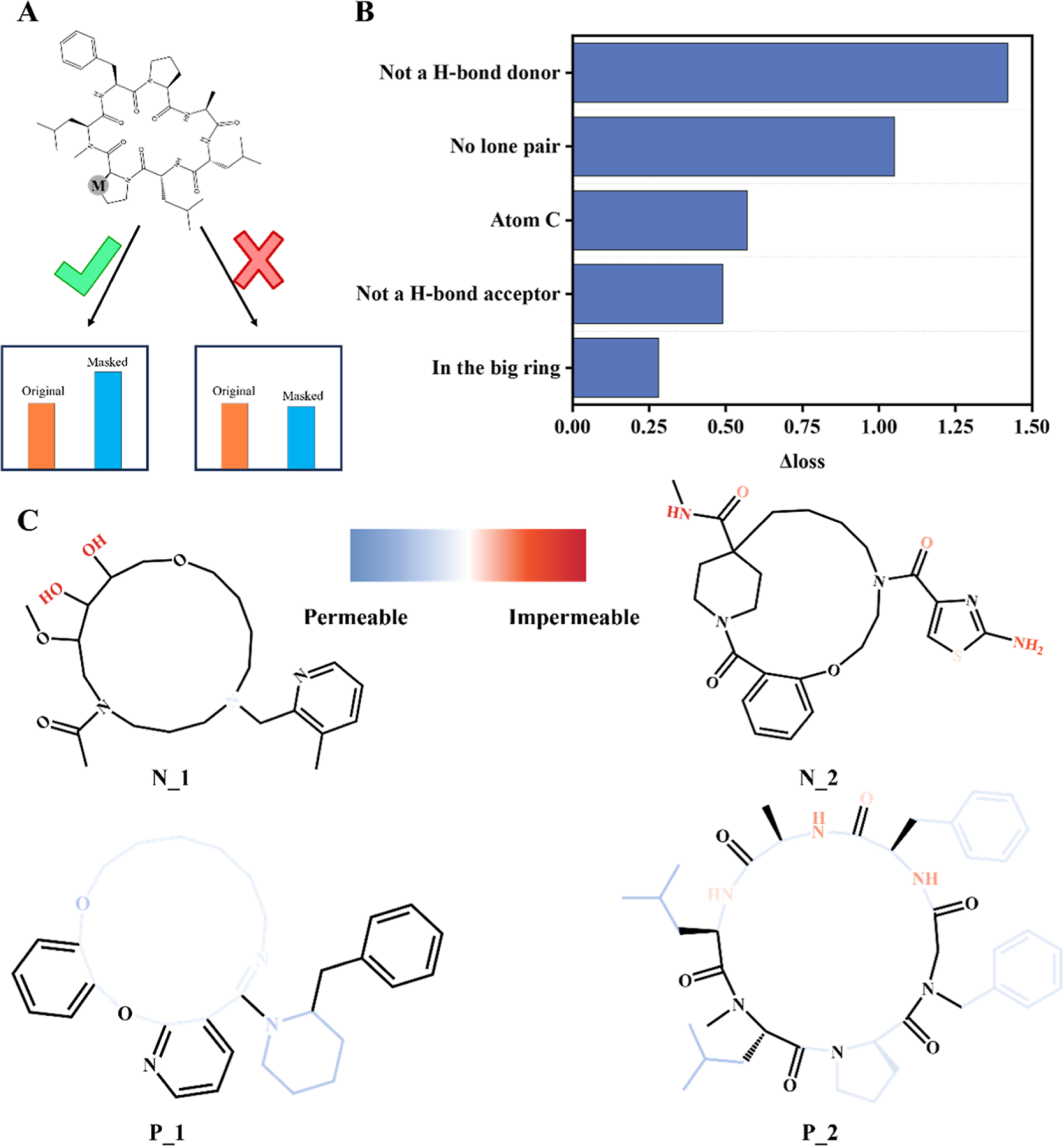
Importance of node features. (A) Node masking strategy is applied to molecular graphs to assess the impact of individual nodes on model performance. (B) Ranking of node feature importance based on performance differences observed with and without masking each feature. (C) Representative structures of two impermeable and two permeable macrocycles illustrate key features influencing permeability, including solvent-exposed polar groups, hydrogen-bond donors, and hydrophobic or ring-embedded motifs.

We further examined four representative compounds (two permeable and two impermeable) to illustrate the substructure-level contributions to permeability. In the permeable cases, N_1 contains two hydroxyl groups acting as strong hydrogen-bond donors on the flexible side chains, leaving the polar functionalities fully exposed to the solvent. Such exposure facilitates intermolecular hydrogen bonding with water, creating a substantial desolvation penalty prior to membrane entry. N_2 similarly presents solvent-exposed polar groups on its side chains (Figure 4C). Notably, these exposed substituents contribute far more to impermeability than the polar groups embedded within the large ring of P_2, illustrating how macrocyclic scaffolds can partially shield the polarity. In contrast, permeable examples P_1 and P_2 highlight structural motifs associated with improved permeability. Ortho-aromatic and alicyclic groups, as well as methyl substituents, increase local hydrophobicity and reduce the effective polarity, thereby facilitating membrane transport. Together, these cases illustrate how side-chain polarity, ring-embedded shielding, and hydrophobic substitution patterns collectively shape macrocycle permeability.

### Top 50 High-Confidence Cases: Linking 2D Graph Features to the 3D Structure

To further explore how substructures affect predictions, we analyzed the 50 most confidently classified permeable (positive) and impermeable (negative) macrocycles. Because single conformations can bias interpretation, we generated an ensemble of 50 conformations per compound and computed average values based on energy and RMSD criteria. Analysis of the 2D topology revealed that negative instances contain more hydrogen-bond donors (HBDs) located on flexible side chains (Figure 5A). Examination of the corresponding 3D structures showed that these side-chain HBDs rarely participate in stabilizing intramolecular hydrogen bonds, whereas HBDs embedded within large rings are more capable of forming internal hydrogen bonds with proximal acceptors—a hallmark of the macrocyclic “chameleon effect.”

**Figure 5:**
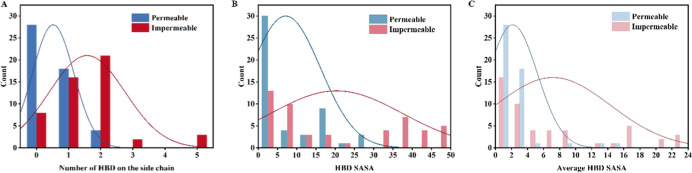
HBD exposure in permeable (positive) and impermeable (negative) macrocycles. (A) Distribution of side-chain HBD counts in positive and negative instances. (B) Distribution of total HBD solvent-accessible surface area (SASA) averaged across 50 conformations. (C) Average per-HBD SASA for positive versus negative macrocycles.

We next computed the SASA of HBDs (average across 50 conformations per molecule). Positive instances showed substantially lower HBD SASA values (Figure 5B), indicating greater shielding of polar groups. Consistently, the average per-donor SASA was significantly lower in permeable compounds (Figure 5C). Permeable examples employed multiple strategies to minimize polarity exposure, including intramolecular hydrogen bonding, ortho-hydrophobic shielding, and steric occlusion of polar groups. We also analyzed HBA SASA (Figure S9A,B) and observed similar trends, although HBD exposure had a stronger influence on model predictions. Overall, these results demonstrate that effective hiding of polar groups—particularly side-chain HBDs—is critical for macrocycle permeability.

### Regression Model

In addition to achieving successful classification on our high-fidelity data set, we also constructed a regression model using the unrestricted data set (10,806 data points). In addition to the physical and chemical properties of molecules, physiological parameters are pivotal in membrane permeability. In our regression model, we used multiple strategies to represent the experimental environment that mimics physiological conditions. These include one-hot encoding physiological conditions, incorporating global features into molecular graph, and extracting key descriptors from textual description using natural language processing. Among these, the representation using global features yielded the best performance. As shown in Figure 6A, the model achieved a coefficient of determination (*R* ^2^) of 0.794 on the training set and 0.741 on the test set. Performance metrics for other representation strategies are summarized in Table S14. Compared with current state-of-the-art regression models for predicting membrane permeability of macrocycles, our approach demonstrates a substantial improvement. Notably, both our classification and regression architectures consistently outperform other DL models.

**Figure 6:**
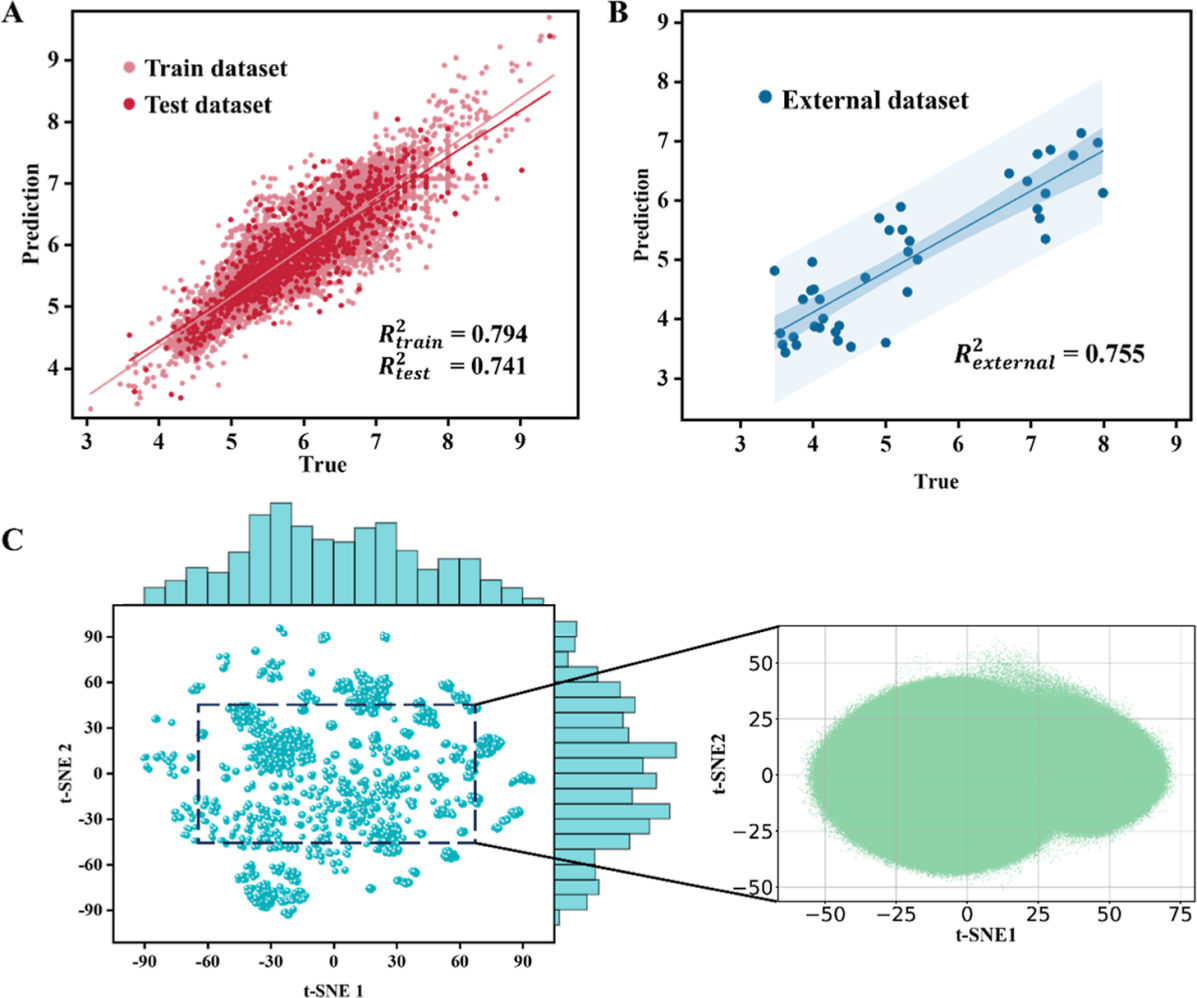
Evaluation of regression models and distribution of the macrocycle library. (A) Evaluation of the regression model on training and test data sets. (B) Evaluation of the regression model on the external data set. (C) Distribution of our data set and macrocycle library.

### External Data Set

To assess the performance of the model on unprecedented data, we evaluated the model on a new external data set which is collected from publications.^42–44^ To comply with the high-fidelity data requirements, only entries meeting strict quality criteria were retained. This external data set contains 40 entries, with the distributions of MW, log *P*, HBD, and HBA summarized in Figure S10A–D. For the classification task, the model achieved strong performance: ACC = 0.950, F1 = 0.963, and AUC = 0.970. For the regression task, the model also performed well, with *R* ^2^ = 0.755 (Figure 6B). The performance on the external data set demonstrates the applicability of the model to unprecedented data. Additionally, we calculated the distribution of the macrocycle library, which comprises approximately 22M small-ringed macrocycles. A comparison with the distribution of the model data set is shown in Figure 6C. The dense coverage of the model data set effectively spans the chemical space of the macrocycle library.

## Discussion and Conclusion

Advances in understanding factors that govern the cell penetrance of macrocycles have not kept pace with discovery methods used to explore the biological function of macrocycles. Accelerating the prediction of permeability is, therefore, critical for macrocycle-based drug discovery. Recent studies include theoretical models based on calculated dynamic molecular surface properties,^34^ an atomistic physical model,^30^ molecular dynamic simulation to identify conformations,^35,45^ and DL models such as Multi_CycGT^36^ (trained on a single cell line) and GNN,^38^ which predict permeability in different cell lines. However, these models either are not generalizable or do not achieve a desirable result due to the sparsity of data.

In this study, we introduce Multi_DDPP, a pretrained DL model leveraging a large data set of related tasks to distill latent knowledge into a task-specific data set. Unlike previous methods that rely on fixed thresholds for labeling,^46^ we introduce a swing range to retain more data and mitigate the experimental variability. By counting experimental results from different research groups for the same macrocyclic molecules (227 pairs), we enhanced the label reliability and prediction accuracy. Multi_DDPP significantly outperforms existing machine learning and DL models across various molecular representations. We also explored the effects of a large data set on the performance of the model after data set distillation. While some improvements were observed, we found that high-fidelity molecules often share similar scaffolds and features with the original data set, limiting the model’s ability to extract novel latent information. Furthermore, we constructed regression models incorporating diverse representations of physiological parameters. For the regression task, the model achieved considerable success, demonstrating that it is essential to consider more comprehensive representations and not just focusing on molecular information.

Multi_DDPP also presents a strong performance with unprecedented data. Although Multi_DDPP has been proven to be efficient in the prediction of permeability of macrocycles, some modifications could still improve the model. For instance, its training relies on the knowledge from the large data set, descriptors, and a molecular graph that focuses on 2D structure information. Future work should explore richer and more detailed molecular representations, such as 3D structural information on the big ring, which compensates for the absence of certain structural information; for different ingredients of the rings, they have different flexibility, electronic environments, and hydrophobicity. In the future, people can combine multimodal representations related to the membrane like simulating a cellular environment by a virtual cell,^47^ enabling more comprehensive permeability predictions in different cellular environments.

## Experimental Section

### Model Architecture

We used knowledge distillation to transfer latent information captured from the large data set. A vector of logits *z* was used to convey information, and the distillation loss is defined as

1 $$L_{D}\left(Z_{B},Z_{S}\right)=L_{D}\left(Z_{B},Z_{S}\right)$$

where $L_{D}\left(\right)$ represents the divergence loss of logits, and *Z*_B_ and *Z*_S_ represent the logits of the large data set and task-specific data set, respectively.

We generate soft targets from the multicell lines data set, which reflect the probabilities of classes to judge the belonging label. These probabilities are calculated using the sigmoid function with temperature scaling:

2 $$p\left(Z_{i},T\right)=\frac{\exp\left(Z_{i}/T\right)}{1+\exp\left(Z_{i}/T\right)}$$

where *z*_*i*_ represents the logit for the prediction, and *T* is a temperature parameter used to adjust the softness of the probability distribution. By these soft labels, the model on the task-specific data set can absorb informative dark knowledge from the multi_cell lines data set. The distillation loss of soft logits can be formulated as

3 $$L_{D}\left(p\left(Z_{B},T\right),p\left(Z_{S},T\right)\right)=L_{D}\left(p\left(Z_{B},T\right),p\left(Z_{S},T\right)\right)$$

where $L_{D}\left(\right)$ represents the divergence loss of logits, *Z*_B_ and *Z*_S_ represent the logits of the large data set and task-specific data set, respectively, and *T* is a temperature parameter.

To balance supervised learning and data distillation, we use a parameter λ to control the weights of dependence on true labels and information from soft labels. This prevents the model from focusing on mimicking the model on the large data set. Accordingly, the loss of model on the task-specific data set can be formulated as

4 $$L_{S}=L_{true}+\lambda L_{soft}$$

where $L_{true}$ represents the binary cross-entropy loss between predicted labels and true labels, $L_{soft}$ is the loss for soft logits, and λ is a parameter to adjust the weights between true labels and soft labels.

### Framework of Macro_PP

It is essential for the model to address diverse and complicated molecular representations, which have direct impacts on the predicted results. We employed an MoE architecture that combines expert networks with a gating mechanism enabling the model to learn fine-grained representations based on different inputs. To be more specific, the input feature $X\in R^{b\times d}$ (b indicates the batch size and d represents the dimension) is allocated into different experts, and *e*_*i*_ is generated through the fully connected network, which can be formulated as

5 $$e_{i}=f_{i}\left(x\right)$$

where *f*_*i*_() represents neural mapping for the *i*-th expert. The output of each expert can be denoted as

6 $$e=\left[\begin{array}{l} e_{1}\\ \cdot \\ e_{N} \end{array}\right]\in R^{b\times N}$$

where *b* is the batch size and *N* represents the number of experts. Then, a weight vector is generated by the gating network that can be calculated by a soft max function:

7 g=softmax(g(x))

where *x* represents the feature input, and *g*(*x*) is the mapping function for gating networks. This is transformed into a probability distribution:

8 $$g_{j}=\frac{e^{g_{j}\left(x\right)}}{\sum_{k=1}^{N}e^{g_{k}\left(x\right)}}$$

where *g*_*j*_ represents the weight of the *j*-th expert, and *g*_*j*_(*x*) represents the correlation score of the *j*-th expert for the input feature. Then, MoE combines the weights of the gating network and the output of the expert network, and we obtain the final output *o* that can be formulated as

9 $$o=\sum_{i=1}^{N}g_{i}e_{i}$$

where *g*_*i*_ and *e*_*i*_ represents the weight matrix and output matrix, respectively.

We used DMPNN to capture the complex connection between nodes and bonds, which can integrate local features through multiple rounds of message passing. To be more specific, SMILES strings are transformed into node and bond features, for example, node feature *X* and bond feature *E* can be denoted as follows:

10 $$X=\left[\begin{array}{l} X_{1}\\ \cdot \\ X_{\left|V\right|} \end{array}\right]$$

11 $$E=\left[\begin{array}{l} e_{1}\\ \cdot \\ e_{\left|E\right|} \end{array}\right]$$

where |*V*| and |*E*| represent the number of nodes and bonds, respectively. Then, MLP is used to generate messages through the bond features between nodes. The message can be formulated as

12 $$m_{uv}=msg\left(e_{uv},X_{u},X_{v}\right)=MLP\left(e_{uv}⊕X_{u}⊕X_{v}\right)$$

where ⊕ represents montage, *e*_uv_ is the bond feature between node u and node *v*, and MLP­() is a series of fully connected layers. Then, messages are aggregated from neighbor nodes:

13 $$m_{v}^{\left(t\right)}=\sum_{u\in \varkappa \left(v\right)}m_{uv}^{\left(t-1\right)}$$

where ϰ­(*v*) is the set of neighbor nodes connected to node *v*, and *t* represents the round of message passing. After message passing, the updated bond feature and node feature can be calculated from as follows:

14 $$e_{uv}^{\left(t\right)}=MLP\left(e_{uv}^{\left(t-1\right)}⊕m_{uv}^{\left(t\right)}\right)$$

15 $$x_{v}^{\left(t\right)}=MLP\left(x_{v}^{\left(t-1\right)}⊕m_{v}^{\left(t\right)}\right)$$

where *e*_*uv*_^(*t*)^, *x*_*v*_^(*t*)^ represents the updated bond feature and node feature, respectively. Finally, local features are aggregated into the global graph feature:

16 $$h_{G}=pool\left(\{x_{v}^{\left(T\right)}:v\in V\}\right)$$

where *x*_*v*_^(T)^ is the feature of node *v* after T rounds of message passing, pool­() represents the pooling operation, and *V* is the set of nodes including all nodes of graph.

We optimized the processing of multiple input features using the gating mechanism to enhance collaboration among experts, which is conducive to improving the model’s understanding of molecular representations.

### Attention Mechanism in the Regression Model

To further capture the interaction between nodes, we used multi-head attention, which introduced the local attention mechanism from the transformers into graph neural networks, achieving more local structural information. Scaled Dot-Product Attention can be formulated as

17 $$Attention\left(Q,K,V\right)=Soft\max\left(\frac{QK^{T}}{\sqrt{d_{k}}}\right)V$$

where *Q*, *K*, and *V* represent query, key, value, respectively, and *d*_*k*_ is the dimension of key.

Multi-head attention can capture more complex local interactions, and it can be represented as

18 $${head}_{i}=Attention\left(Q_{i},K_{i},V_{i}\right)$$

19 $$Multi\;Head\left(Q,K,V\right)=Concact\left({head}_{1},{head}_{2},\cdot \cdot \cdot ,{head}_{i}\right)W^{O}$$

where *W* ^O^ is the projection matrix including the information on each head.

### Multiple Cell Lines Data Set

We collected permeability data from widely used cell lines (Caco-2, MDCK, RRCK, and PAMPA) via CHEMBL and PubChem. Duplicates (e.g., tested under different assay conditions) were removed by using InChiKey. The final data set includes 23,086 entries covering small molecules, linear peptides, and macrocycles. The statistics for each assay and different types of molecules are shown in Table S15. For the Caco-2 assay, we followed established permeability classifications, in which compounds with P__app_ < 1 × 10^–6^ cm/s, 1–10 × 10^–6^ cm/s, and >10 × 10^–6^ cm/s correspond to poorly (0–20%), moderately (20–70%), and well-absorbed (70–100%) compounds, respectively.^48^ For MDCK and RRCK assays, we applied the same thresholding scheme, as both cell lines are widely used as Caco-2 surrogates for passive permeability screening.^49,50^ To retain as much data as possible, we split positive and negative samples by using a unified threshold (−log *P* = 6 as a threshold). The positive samples account for 75%. To avoid possible data bias problems, 10-fold cross-validation was used to split the data set. We also used the unique SMILES string to split the data set, avoiding data leakage, and indexed it through the unique SMILES string, ensuring no duplicates in the training set and validation set.

### High-Fidelity Task-specific Data Set

We collected task-specific (PAMPA permeability of macrocycles) data from CycPeptMPDB,^46^ NPMMPD,^51^ and literature. Then, we collected 227 macrocycles tested by different research groups. A reliable and rigorous data set is essential for model construction. We counted the range of deviations and set a swing range (5.5←log *P* < 6.5), which not only effectively mitigates errors arising from experimental conditions but also maximizes the retention of most data. Compared with the general split method (the −log *P* value ≥ 6.0 as positive samples, <6.0 as negative samples), a swing range provides a tolerance for experimental errors, alleviating the label noise. After removing duplicates via InChIKey, we obtained a high-fidelity data set of 6733 entries (3999 permeable/positive, 2734 impermeable/negative). We used 10-fold cross-validation to avoid data bias.

### Data Set for Regression Models

Unlike classification tasks, the regression data set did not require labeling. All of the data were retained. Physiological parameters from the literature (e.g., pH, temperature) were extracted from experimental descriptions in the literature.

### Nodes and Edges of the Molecular Graph

Nodes and edges of the molecular graph are clarified in Table S16.

### Hyperparameters of Multi_DDPP

Hyperparameters of Multi_DDPP are clarified in Table S17.

### Baselines

We used different molecular representations including descriptors (Mordred,^52^ Rdkit), fingerprints (ECFP, MACCS), molecular graph, and the combination of different representations as input features. Here, we constructed different machine learning and DL models based on these representations to compare with our model Multi_DDPP: descriptors-based models (Mordred_RF, Mordred_SVM, Mordred_XGB, Mordred_GBDT, Rdkit_RF, Rdkit_SVM, Rdkit_XGB, Rdkit_GBDT), fingerprints-based models (ECFP_RF, ECFP_SVM, ECFP_XGB, ECFP_GBDT, MACCS_RF, MACCS_SVM, MACCS_XGB, MACCS_GBDT), graph-based models (AttentiveFP,^53^ GAT,^54^ GCN,^55^ InfoGraph,^56^ MPNN,^57^ DMPNN^58^), pretrained models (ChemBERTa-3,^59^ Uni-mol^60^), and combined representations-based models (Chemprop,^61^ Macro_PP).

### Evaluation Metrics

The AUC, MCC, accuracy (ACC), and binary cross-entropy loss are used for comprehensive evaluation:

20 $$ACC=\frac{TP+TN}{TP+TN+FP+FN}$$

21 $$MCC=\frac{TP\times TN-FP\times FN}{\sqrt{\left(TP+FP\right)\left(TP+FN\right)\left(TN+FP\right)\left(TN+FN\right)}}$$

22 $$L_{BCE}=-\frac{1}{N}\sum_{i=1}^{N}y_{i}\log\left(p\left(y_{i}\right)\right)+\left(1-y_{i}\right)\log\left(1-p\left(y_{i}\right)\right)$$

where TP, TN, FP, and FN are the true positive, true negative, false positive, and false negative, respectively, which are used to represent contrast between the true and the predicted. *y*_*i*_ is a binary label, *p*(*y*_*i*_) represents the probability of the label, and *N* is the number of predicted samples.

### Identification of Intramolecular Hydrogen Bonds

Intramolecular hydrogen bonds (IMHBs) were identified using standard geometric criteria combining distance and angular constraints. These criteria follow established hydrogen-bond definitions.^62^ The distance between the hydrogen atom (H) on HBD and the acceptor atom (A) and the angle formed by the donor (D), hydrogen (H), and acceptor (A) atoms should satisfy the following:

23 d(H···A)≤2.5Å

24 $$∠\left(D\cdot \cdot \cdot H\cdot \cdot \cdot A\right)\geq 120^{{}^{\circ}}$$

#### SASA of Polar Groups

Solvent-accessible surface areas (SASAs) were computed using the Shrake*–*Rupley algorithm.^63,64^ For each atom *i*, the SASA was calculated as

25 $${SASA}_{i}=4\pi {\left(R_{i}+R_{probe}\right)}^{2}\times \left(\frac{N_{accessible,i}}{N_{total,i}}\right)$$

where *R*_*i*_ is the van der Waals radius of atom *i*, *R*_probe_ is the probe radius (1.4 Å for water), *N*_accessible,*i*_ represents the number of accessible test points, and *N*_total,*i*_ is the total number of test points placed around the atomic sphere.

## Supplementary Material



## Data Availability

Multi_DDPP, regression models, and data sets are available at https://github.com/YUZhang-utu/Macro_permeability.

## Notes

Model performances (DL models and machine learning models) in different-level noises, distribution of data in the big data set, evaluation of graph-only models, evaluation of regression models, description of nodes, edges, and descriptors, hyperparameters of models, distribution of data based on the size of ring in different-level noises, evaluation of models on different sizes of the ring, and data distribution of the external data set (PDF)

## Abbreviations

ACC: accuracy
ADME: absorption, distribution, metabolism, excretion
AUC: area under the curve
DL: deep learning
DMPNN: directed message passing neural network
FDA: Food and Drug Administration
FN: false negative
GAT: graph attention network
GBDT: gradient boosting decision tree
GCN: graph convolutional network
HBA: hydrogen bond acceptor
HBD: hydrogen bond donor
MAE: mean absolute error
MCC: Matthews correlation coefficient
MDCK: Madin–Darby canine kidney
MPNN: message passing neural network
ML: machine learning
PAMPA: parallel artificial membrane permeability assay
*R* ^2^: coefficient of determination
RF: random forest
RMSE: root-mean-squared error
RRCK: Ralph Russ canine kidney
SASA: solvent-accessible surface area
SVM: support vector machine
TN: true negative
TP: true positive
t-SNE: t-distributed stochastic neighbor embedding
XGB: eXtreme gradient boosting.
