# Supplementary material for: Enhancing the Predictive Power of Macrocyclic Drug Permeability by Knowledge Distillation from Analogous Pretraining Data
Source: J Med Chem. 2025 Dec 20;69(1):387–99. doi: 10.1021/acs.jmedchem.5c02620 (PMC12794141; doi:10.1021/acs.jmedchem.5c02620)
Supplement: Supplementary file 1 [file jm5c02620_si_001.pdf]

Supporting Information

Enhancing the Predictive Power of Macrocyclic Drug Permeability by Knowledge Distillation from Analogous Pre-training Data

Yu Zhang[1,2,] *, Olli T. Pentikäinen[1,2,] *

1. Institute of Biomedicine, Integrative Physiology and Pharmacy, University of Turku, FI-20014 Turku, Finland
2. InFLAMES Research Flagship Center, University of Turku, FI-20014 Turku, Finland

*Corresponding Authors:
Yu Zhang: yuzhang@utu.fi
Olli T. Pentikäinen: olli.pentikainen@utu.fi

Table of contents

Table S1. Mordred descriptors. S3
Table S2. Baseline machine learning models (swing value=0.5). S3
Table S3. Baseline deep learning models, Macro_PP, and Multi_DDPP (swing value=0.5). S4
Table S4. Performance of Macro_PP and Multi_DDPP based on Murcko-scaffold split S4
Table S5. Baseline deep learning models, Macro_PP, and Multi_DDPP (swing value=0.4). S4
Table S6. Baseline deep learning models, Macro_PP, and Multi_DDPP (swing value=0.6). S4
Table S7. Baseline machine learning models (swing value=0.2). S5
Table S8. Baseline machine learning models (swing value=0.4). S5
Table S9. Baseline machine learning models (swing value=0.6). S6
Table S10. Baseline machine learning models (swing value=0.8). S6
Table S11. Evaluation of models (percentage of added high-fidelity data). S7
Table S12. Evaluation of models based on different methods of adding high-fidelity data (different percentages). S7
Table S13. Ablation experiment (graph-only Macro_PP and Multi_DDPP) S8
Table S14. Evaluation of regression models. S8
Table S15. Statistics of data in the big dataset S8
Table S16. Features of nodes and edges S8
Table S17. Hyperparameters of the model S9
Figure S1. The distribution of data with different bRO5 numbers. S9
Figure S2. Data distribution in different swing values S10
Figure S3. Counts of data based on ring size in different datasets with different swing values S10
Figure S4. Performance of Macro_PP and Multi_DDPP in different ring size datasets S11
Figure S5. Using Elbow method and Silhouette scores to get the clusters of clustering. S12
Figure S6. The evaluation of pre-trained models with different percentages of added data. S12
Figure S7. A, The distribution of added data based on fingerprint method. **B**, The distribution of added data randomly. **C**, The distribution of added data based on scaffold method. S13
Figure S8. The evaluation of models based on three ways (fingerprint, random, scaffold) to select different percentages of added data. S14
Figure S9. A, The distribution of HBA SASA. B, The distribution of average HBA SASA S14
Figure S10. A, The distribution of HBA SASA. B, The distribution of average HBA SASA S15

Table S1. Mordred descriptors.

Descriptors	Description
AATS0m	autocorrelation of lag 0 weighted by mass
AATS1v	autocorrelation of lag 1 weighted by vdw volume
AATS1i	autocorrelation of lag 1 weighted by ionization potential
ATSC1Z	autocorrelation of lag 1 weighted by atomic number
ATSC2v	autocorrelation of lag 2 weighted by vdw volume
ATSC3se	autocorrelation of lag 3 weighted by sanderson EN
ATSC5se	autocorrelation of lag 5 weighted by sanderson EN
ATSC6pe	autocorrelation of lag 6 weighted by pauling EN
ATSC8are	autocorrelation of lag 8 weighted by allred-rocow EN
SssCH2	sum of ssCH2
MIC0	0-ordered modified information content
MIC2	2-ordered modified information content
PEOE_VSA1	MOE Charge VSA Descriptor 1 (-inf < x < -0.30)
PEOE_VSA6	MOE Charge VSA Descriptor 6 (-0.10 <= x < -0.05)
EState_VSA10	EState VSA Descriptor 10 (9.17 <= x < 15.00)
VSA_EState3	VSA EState Descriptor 3 (5.00 <= x < 5.41)
nRot	rotatable bonds count
SLogP	Wildman-Crippen LogP
Diameter	topological diameter

Table S2. Baseline machine learning models (swing value=0.5).

	ACC	AUC	MCC
RF_Mordred	0.889±0.009	0.945±0.009	0.767±0.021
SVM_ Mordred	0.877±0.012	0.938±0.008	0.744±0.026
XGB_Mordred	0.895±0.011	0.956±0.007	0.782±0.023
GBDT_Mordred	0.880±0.012	0.949±0.006	0.750±0.028
RF_Rdkit	0.888±0.011	0.945±0.009	0.766±0.023
SVM_Rdkit	0.854±0.014	0.922±0.008	0.695±0.032
XGB_Rdkit	0.885±0.013	0.949±0.007	0.759±0.028
GBDT_Rdkit	0.864±0.014	0.934±0.010	0.716±0.030
RF_MACCS	0.845±0.009	0.924±0.008	0.677±0.017
SVM_MACCS	0.815±0.014	0.884±0.014	0.615±0.031
XGB_MACCS	0.844±0.011	0.923±0.008	0.676±0.019
GBDT_MACCS	0.799±0.013	0.884±0.012	0.582±0.025
RF_ECFP	0.884±0.010	0.945±0.008	0.757±0.022
SVM_ECFP	0.875±0.010	0.938±0.006	0.739±0.022
XGB_ECFP	0.889±0.014	0.953±0.007	0.770±0.030
GBDT_ECFP	0.851±0.017	0.923±0.011	0.689±0.039

Table S3. Baseline deep learning models, Macro_PP, and Multi_DDPP (swing value=0.5).

	ACC	AUC	MCC
AttentiveFP	0.899±0.012	0.955±0.008	0.791±0.025
GAT	0.866±0.013	0.937±0.010	0.721±0.028
GCN	0.855±0.024	0.939±0.011	0.701±0.048
InfoGraph	0.839±0.019	0.914±0.016	0.673±0.035
MPNN	0.888±0.012	0.941±0.009	0.769±0.026
Chemprop	0.902±0.010	0.959±0.004	0.797±0.019
DMPNN	0.904±0.009	0.952±0.008	0.803±0.021
ChemBERTa-3	0.898±0.010	0.949±0.008	0.788±0.020
Uni-mol	0.886±0.011	0.947±0.008	0.763±0.023
Macro_PP	0.912±0.008	0.964±0.005	0.818±0.019
Multi_DDPP	0.948±0.010	0.988±0.008	0.892±0.022

Table S4. Performance of Macro_PP and Multi_DDPP based on Murcko-scaffold split

	ACC	AUC	MCC	PR-AUC
Macro_PP	0.890±0.017	0.939±0.012	0.765±0.037	0.956±0.011
Multi_DDPP	0.933±0.016	0.978±0.011	0.857±0.033	0.982±0.009

Table S5. Baseline deep learning models, Macro_PP, and Multi_DDPP (swing value=0.4).

	ACC	AUC	MCC	PR-AUC
AttentiveFP	0.894±0.009	0.949±0.009	0.781±0.018	0.958±0.009
GAT	0.840±0.016	0.914±0.019	0.666±0.033	0.934±0.016
GCN	0.855±0.024	0.937±0.010	0.702±0.046	0.952±0.008
InfoGraph	0.792±0.028	0.800±0.021	0.591±0.045	0.808±0.016
MPNN	0.885±0.011	0.940±0.006	0.760±0.024	0.948±0.008
Chemprop	0.894±0.008	0.954±0.005	0.783±0.017	0.964±0.005
DMPNN	0.899±0.010	0.952±0.006	0.792±0.019	0.956±0.008
ChemBERTa-3	0.890±0.012	0.939±0.010	0.770±0.026	0.948±0.009
Uni-mol	0.879±0.012	0.963±0.007	0.748±0.026	0.951±0.011
Macro_PP	0.905±0.006	0.959±0.006	0.803±0.014	0.968±0.008
Multi_DDPP	0.938±0.012	0.983±0.010	0.872±0.025	0.988±0.009

Table S6. Baseline deep learning models, Macro_PP, and Multi_DDPP (swing value=0.6).

	ACC	AUC	MCC	PR-AUC
AttentiveFP	0.926±0.014	0.969±0.008	0.844±0.032	0.974±0.015

GAT	0.897±0.013	0.958±0.011	0.783±0.028	0.971±0.009
GCN	0.896±0.020	0.965±0.009	0.782±0.045	0.977±0.007
InfoGraph	0.846±0.025	0.837±0.025	0.681±0.040	0.844±0.024
MPNN	0.911±0.013	0.964±0.011	0.812±0.027	0.971±0.014
Chemprop	0.931±0.011	0.977±0.007	0.857±0.024	0.982±0.010
DMPNN	0.929±0.013	0.968±0.010	0.851±0.027	0.971±0.015
ChemBERTa-3	0.920±0.011	0.967±0.005	0.832±0.024	0.973±0.006
Uni-mol	0.908±0.013	0.963±0.007	0.805±0.027	0.974±0.007
Macro_PP	0.937±0.007	0.978±0.006	0.866±0.015	0.983±0.008
Multi_DDPP	0.964±0.014	0.992±0.006	0.925±0.027	0.995±0.004

Table S7. Baseline machine learning models (swing value=0.2).

	ACC	AUC	MCC	PR-AUC
RF_Mordred	0.850±0.015	0.920±0.012	0.684±0.033	0.941±0.011
SVM_ Mordred	0.840±0.011	0.903±0.010	0.664±0.026	0.924±0.008
XGB_Mordred	0.843±0.015	0.915±0.011	0.670±0.033	0.937±0.009
GBDT_Mordred	0.833±0.014	0.908±0.011	0.647±0.032	0.931±0.010
RF_Rdkit	0.845±0.012	0.917±0.011	0.674±0.026	0.939±0.010
SVM_Rdkit	0.817±0.012	0.885±0.012	0.613±0.028	0.914±0.010
XGB_Rdkit	0.825±0.012	0.900±0.011	0.630±0.025	0.927±0.008
GBDT_Rdkit	0.820±0.011	0.893±0.011	0.619±0.025	0.919±0.009
RF_MACCS	0.782±0.006	0.858±0.012	0.544±0.018	0.887±0.008
SVM_MACCS	0.782±0.007	0.827±0.012	0.537±0.019	0.857±0.016
XGB_MACCS	0.773±0.007	0.847±0.012	0.519±0.026	0.882±0.010
GBDT_MACCS	0.771±0.009	0.843±0.014	0.516±0.029	0.878±0.012
RF_ECFP	0.803±0.013	0.879±0.014	0.588±0.028	0.907±0.009
SVM_ECFP	0.813±0.016	0.883±0.012	0.606±0.036	0.910±0.009
XGB_ECFP	0.806±0.012	0.878±0.011	0.591±0.026	0.908±0.009
GBDT_ECFP	0.804±0.012	0.871±0.014	0.587±0.028	0.901±0.011

Table S8. Baseline machine learning models (swing value=0.4).

	ACC	AUC	MCC	PR-AUC
RF_Mordred	0.878±0.010	0.944±0.008	0.745±0.021	0.959±0.006
SVM_ Mordred	0.865±0.012	0.927±0.010	0.717±0.027	0.943±0.008
XGB_Mordred	0.874±0.012	0.942±0.008	0.736±0.027	0.957±0.006
GBDT_Mordred	0.865±0.015	0.935±0.009	0.716±0.032	0.952±0.007
RF_Rdkit	0.872±0.012	0.942±0.008	0.732±0.026	0.956±0.005
SVM_Rdkit	0.841±0.015	0.910±0.012	0.667±0.035	0.933±0.009
XGB_Rdkit	0.853±0.011	0.925±0.010	0.693±0.024	0.945±0.006
GBDT_Rdkit	0.850±0.008	0.921±0.008	0.686±0.020	0.940±0.005
RF_MACCS	0.805±0.012	0.886±0.011	0.594±0.027	0.910±0.009
SVM_MACCS	0.803±0.015	0.872±0.017	0.587±0.032	0.891±0.017

XGB_MACCS	0.796±0.012	0.875±0.015	0.572±0.029	0.901±0.015
GBDT_MACCS	0.789±0.013	0.870±0.015	0.556±0.029	0.895±0.015
RF_ECFP	0.878±0.008	0.944±0.005	0.745±0.019	0.958±0.004
SVM_ECFP	0.834±0.017	0.910±0.014	0.651±0.039	0.932±0.009
XGB_ECFP	0.857±0.013	0.927±0.007	0.700±0.030	0.946±0.005
GBDT_ECFP	0.831±0.012	0.900±0.009	0.647±0.029	0 .922±0.008

Table S9. Baseline machine learning models (swing value=0.6).

	ACC	AUC	MCC	PR-AUC
RF_Mordred	0.895±0.010	0.959±0.008	0.776±0.023	0.972±0.006
SVM_ Mordred	0.893±0.017	0.954±0.009	0.772±0.040	0.967±0.006
XGB_Mordred	0.899±0.010	0.962±0.006	0.786±0.022	0.975±0.005
GBDT_Mordred	0.889±0.012	0.956±0.007	0.766±0.025	0.970±0.006
RF_Rdkit	0.890±0.020	0.955±0.010	0.768±0.044	0.970±0.006
SVM_Rdkit	0.865±0.018	0.935±0.013	0.714±0.040	0.959±0.008
XGB_Rdkit	0.893±0.015	0.957±0.010	0.773±0.034	0.972±0.007
GBDT_Rdkit	0.875±0.022	0.944±0.013	0.734±0.047	0.961±0.009
RF_MACCS	0.846±0.009	0.920±0.013	0.676±0.025	0.940±0.012
SVM_MACCS	0.838±0.013	0.907±0.017	0.654±0.030	0.928±0.019
XGB_MACCS	0.847±0.008	0.929±0.011	0.675±0.023	0.950±0.011
GBDT_MACCS	0.813±0.009	0.901±0.012	0.604±0.020	0.928±0.014
RF_ECFP	0.888±0.014	0.952±0.008	0.762±0.032	0.968±0.007
SVM_ECFP	0.869±0.022	0.939±0.017	0.723±0.049	0.957±0.013
XGB_ECFP	0.896±0.008	0.955±0.009	0.779±0.019	0.969±0.008
GBDT_ECFP	0.854±0.013	0.922±0.010	0.690±0.027	0.942±0.011

Table S10. Baseline machine learning models (swing value=0.8).

	ACC	AUC	MCC	PR-AUC
RF_Mordred	0.909±0.015	0.973±0.010	0.815±0.027	0.978±0.012
SVM_ Mordred	0.906±0.018	0.967±0.010	0.808±0.034	0.975±0.012
XGB_Mordred	0.903±0.014	0.971±0.006	0.801±0.027	0.977±0.008
GBDT_Mordred	0.894±0.012	0.966±0.006	0.783±0.024	0.974±0.009
RF_Rdkit	0.899±0.025	0.968±0.011	0.793±0.049	0.975±0.012
SVM_Rdkit	0.866±0.018	0.947±0.014	0.725±0.037	0.961±0.014
XGB_Rdkit	0.882±0.026	0.958±0.013	0.758±0.051	0.967±0.014
GBDT_Rdkit	0.881±0.030	0.953±0.014	0.756±0.059	0.964±0.013
RF_MACCS	0.857±0.025	0.938±0.013	0.709±0.050	0.946±0.015
SVM_MACCS	0.879±0.010	0.942±0.011	0.753±0.022	0.947±0.016
XGB_MACCS	0.879±0.018	0.944±0.013	0.753±0.037	0.953±0.014
GBDT_MACCS	0.852±0.022	0.925±0.012	0.698±0.040	0.934±0.014
RF_ECFP	0.891±0.016	0.956±0.009	0.778±0.033	0.963±0.010
SVM_ECFP	0.899±0.016	0.962±0.006	0.793±0.033	0.969±0.009

XGB_ECFP	0.905±0.024	0.962±0.009	0.805±0.047	0.968±0.011
GBDT_ECFP	0.894±0.019	0.950±0.011	0.782±0.037	0.956±0.015

Table S11. Evaluation of models (percentage of added high-fidelity data).

	Loss	ACC	AUC	MCC
0	0.407±0.010	0.817±0.005	0.844±0.012	0.451±0.021
10	0.406±0.009	0.820±0.008	0.846±0.007	0.470±0.021
20	0.403±0.013	0.822±0.007	0.850±0.013	0.486±0.032
30	0.398±0.010	0.824±0.007	0.858±0.009	0.505±0.025
40	0.397±0.011	0.827±0.007	0.860±0.011	0.517±0.022
50	0.392±0.014	0.831±0.008	0.865±0.011	0.535±0.030
60	0.387±0.010	0.835±0.005	0.871±0.008	0.552±0.012
70	0.381±0.013	0.837±0.008	0.876±0.010	0.567±0.022
80	0.377±0.012	0.841±0.007	0.882±0.011	0.580±0.024
90	0.373±0.012	0.840±0.005	0.882±0.011	0.580±0.018
100	0.370±0.011	0.844±0.007	0.886±0.009	0.593±0.018

Table S12. Evaluation of models based on different methods of adding high-fidelity data (different percentages).

	ACC	AUC	MCC
10_M	0.949±0.007	0.988±0.006	0.896±0.016
10_R	0.948±0.008	0.988±0.005	0.892±0.019
10_S	0.948±0.008	0.988±0.006	0.893±0.018
20_M	0.949±0.008	0.987±0.006	0.893±0.019
20_R	0.952±0.007	0.988±0.006	0.900±0.016
20_S	0.947±0.010	0.986±0.007	0.890±0.022
30_M	0.950±0.008	0.989±0.005	0.896±0.017
30_R	0.950±0.008	0.988±0.007	0.896±0.018
30_S	0.947±0.008	0.987±0.008	0.890±0.019
40_M	0.950±0.007	0.988±0.006	0.896±0.018
40_R	0.948±0.010	0.986±0.007	0.892±0.022
40_S	0.951±0.008	0.988±0.006	0.899±0.017
50_M	0.952±0.007	0.989±0.005	0.900±0.015
50_R	0.948±0.008	0.987±0.006	0.891±0.018
50_S	0.948±0.008	0.987±0.008	0.893±0.017
60_M	0.947±0.009	0.988±0.006	0.891±0.021
60_R	0.950±0.008	0.988±0.007	0.896±0.019
60_S	0.949±0.009	0.988±0.007	0.896±0.021
70_M	0.950±0.008	0.989±0.007	0.898±0.017
70_R	0.953±0.007	0.989±0.006	0.900±0.015
70_S	0.948±0.008	0.987±0.007	0.893±0.018
80_M	0.948±0.008	0.988±0.006	0.893±0.017

80_R	0.950±0.008	0.988±0.007	0.897±0.018
80_S	0.949±0.007	0.988±0.006	0.894±0.015

Table S13. Ablation experiment (graph-only Macro_PP and Multi_DDPP)

	ACC	AUC	MCC	PR-AUC
Macro_PP	0.904±0.011	0.963±0.007	0.801±0.025	0.969±0.010
Multi_DDPP	0.937±0.012	0.984±0.007	0.869±0.028	0.988±0.009

Table S14. Evaluation of regression models.

	Oh_model[a]	Txt_model[b]	G_model[c]	No_model[d]
Loss (MSE)	0.188	0.220	0.163	0.192
R^2	0.728	0.651	0.741	0.722
r	0.853	0.807	0.861	0.850

[a] Oh_model: one-hot encoding to represent physiological parameters. [b] Txt_model: Nature language processing to extract key words about physiological parameters. [c] G_model: Physiological parameters as global features in the graph. [d] No_model: No physiological parameters.

Table S15. Statistics of data in the big dataset

	PAMPA	Caco-2	MDCK	RRCK
Small molecule	7413	7562	2493	3
Macrocycle	3706	1597	53	0
Linear peptide	124	135	0	0

Table S16. Features of nodes and edges

	Feature
Node	element of the atom
	degree of the atom
	formal charge of the atom
	hybridization of the atom
	number of Hs (explicit and implicit) on the atom
	number of lone electron pairs
	h bond donor
	h bond acceptor
	electronegativity of the atom
	size of the ring containing the atom
	uncommon values beyond the definition
	the type of the bond (Single)
	the type of the bond (Double)

Edge	the type of the bond (Triple)<br>the type of the bond (Aromatic)<br>whether or not the bond is conjugated<br>whether or not the bond is in a ring<br>the stereo configuration of the bond

Table S17. Hyperparameters of the model

Hyperparameters	Settings
Learning rate	$1e^{-4}$
Number of experts in MOE	4
Dropout	0.2
Epoch	300
Batch size	24
Distillation weight (λ)	0.2
Temperature (T)	6.0
Message passing rounds	7

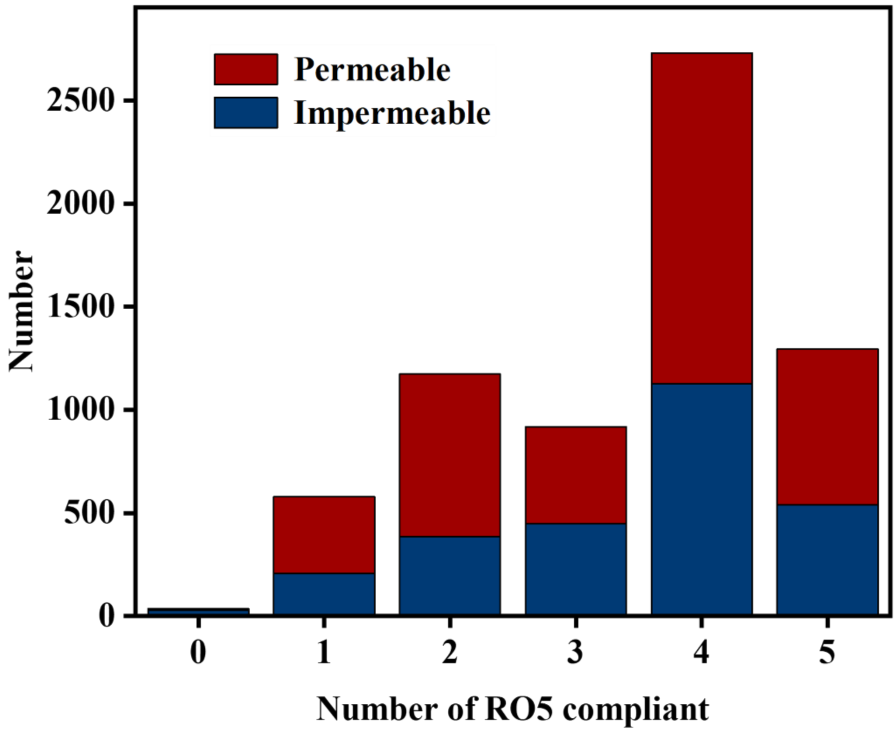

Figure S1. The distribution of data with different bRO5 numbers.

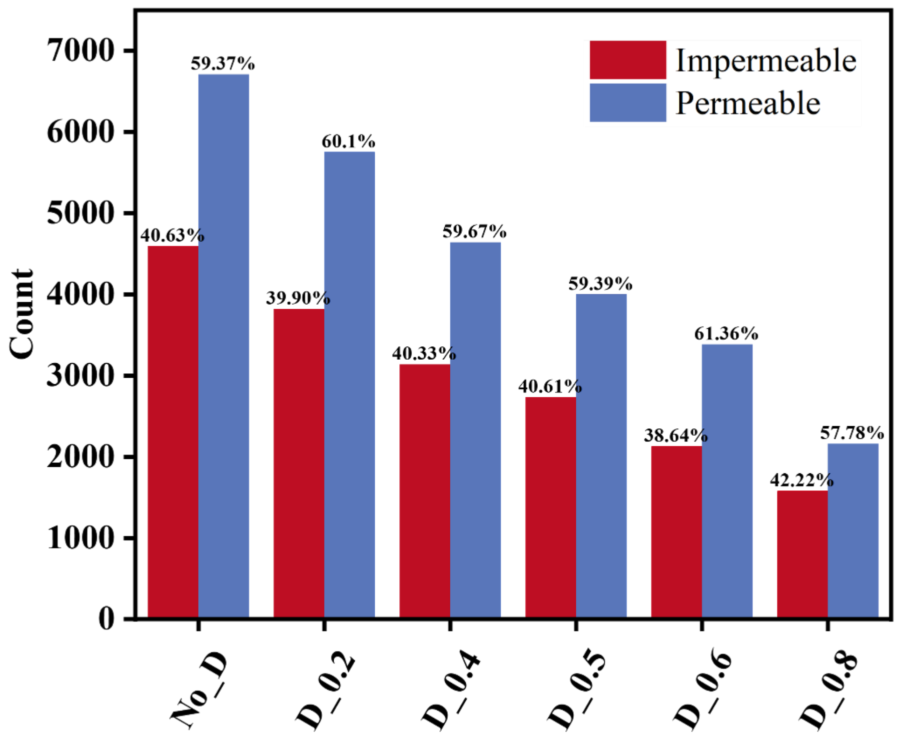

Figure S2. Data distribution in different swing values

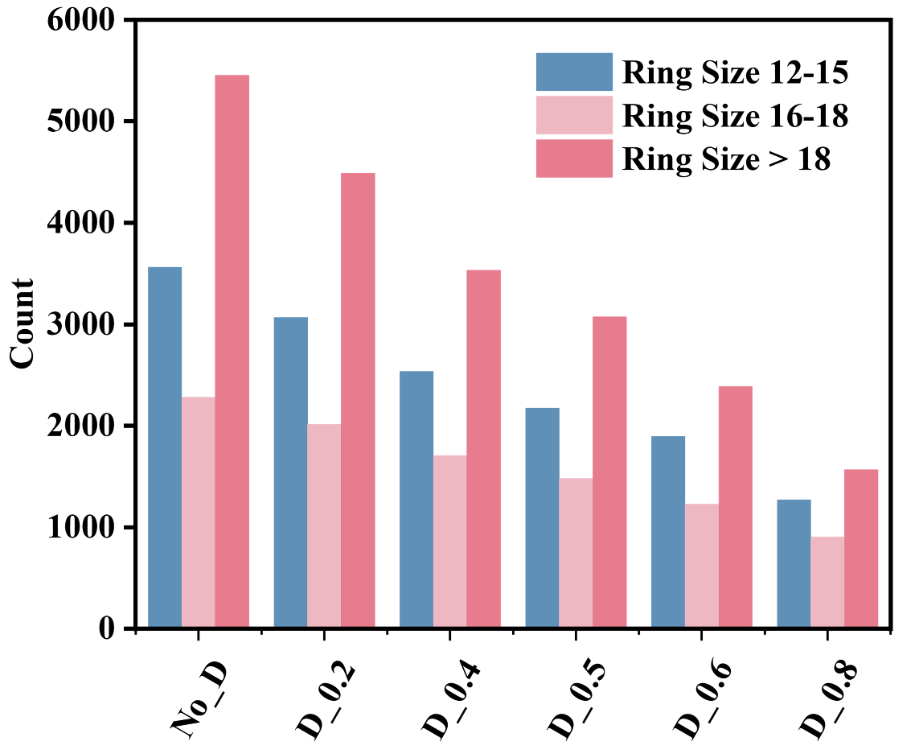

Figure S3. Counts of data based on ring size in different datasets with different swing values

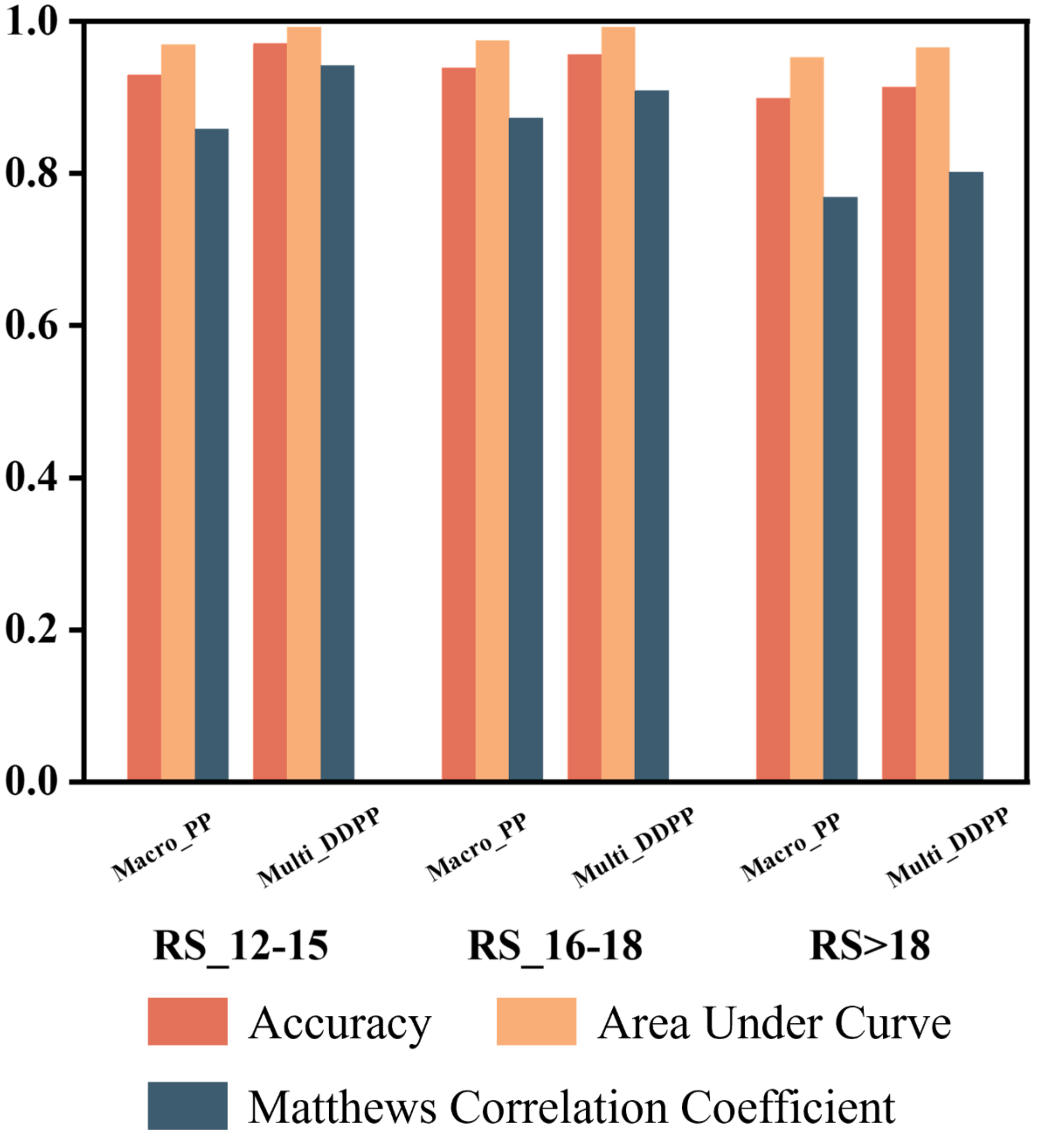

Figure S4. Performance of Macro_PP and Multi_DDPP in different ring size datasets

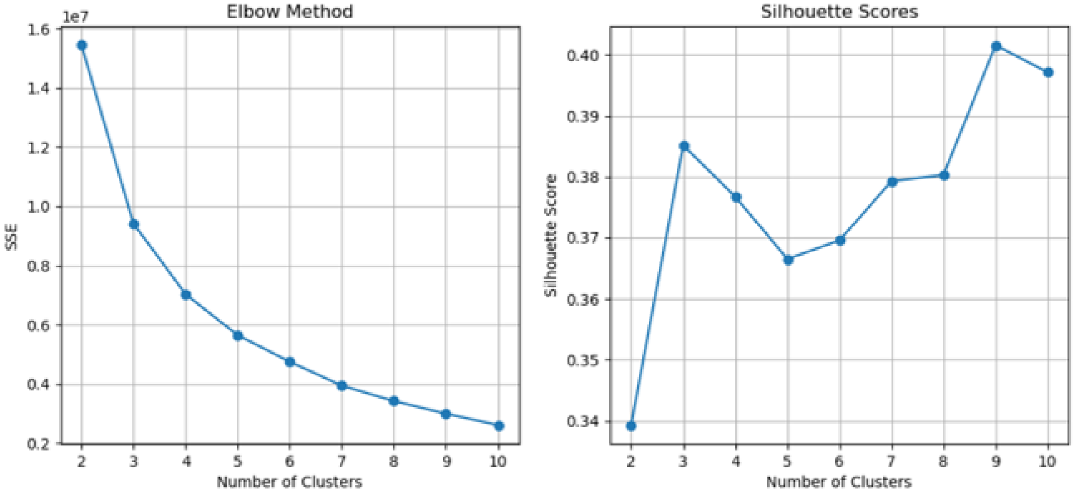

Figure S5. Using Elbow method and Silhouette scores to get the clusters of clustering.

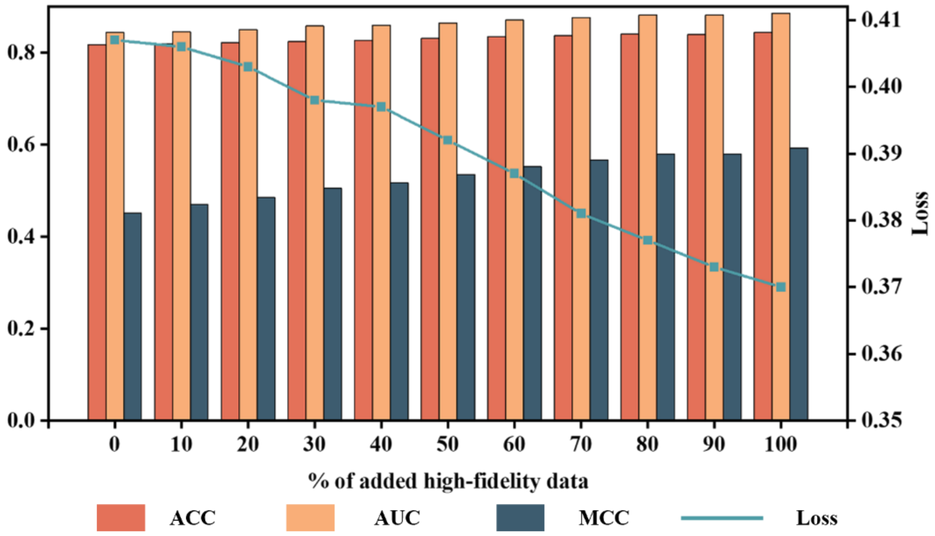

Figure S6. The evaluation of pre-trained models with different percentages of added data.

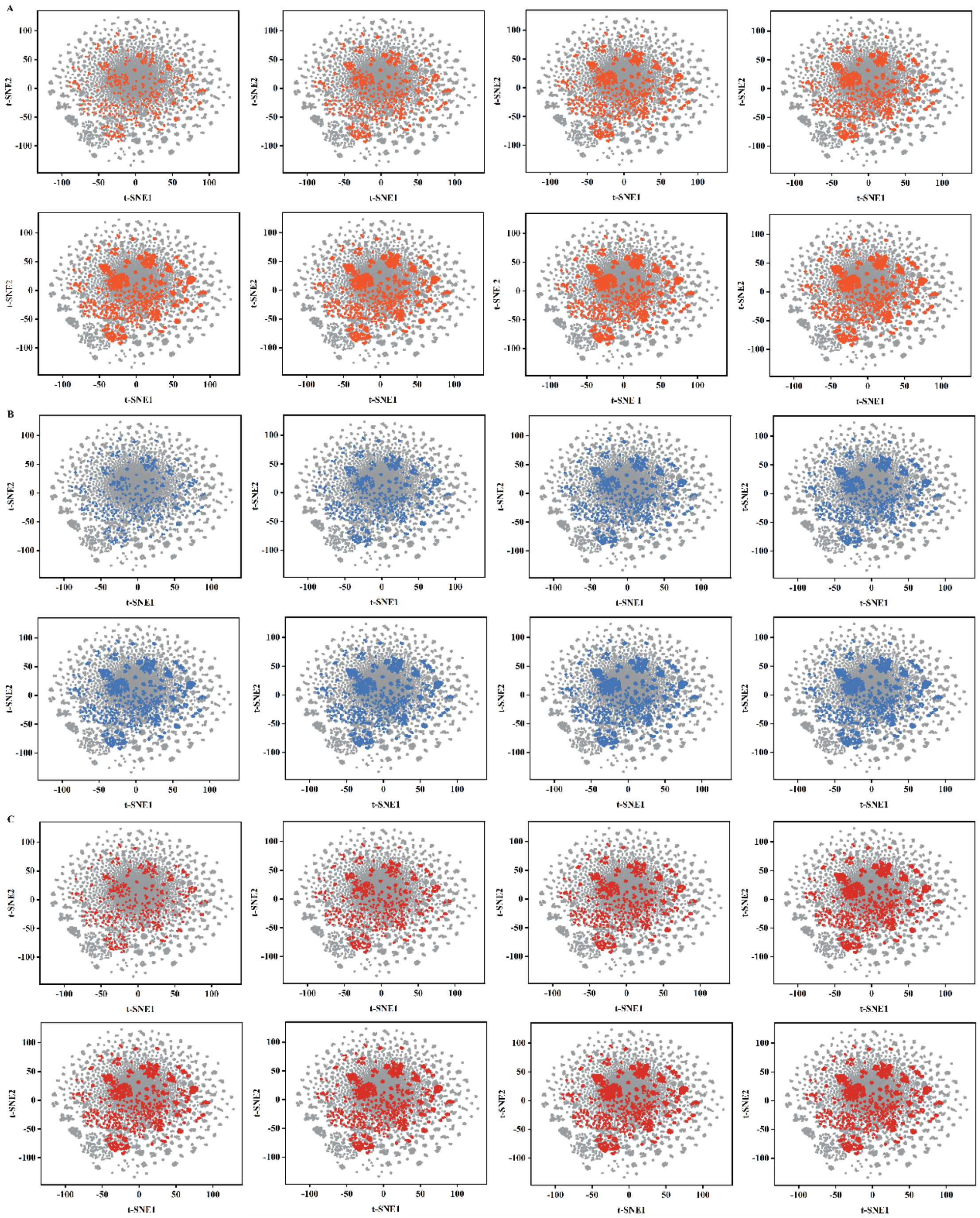

Figure S7. A, The distribution of added data based on fingerprint method. **B**, The distribution of added data randomly. **C**, The distribution of added data based on scaffold method.

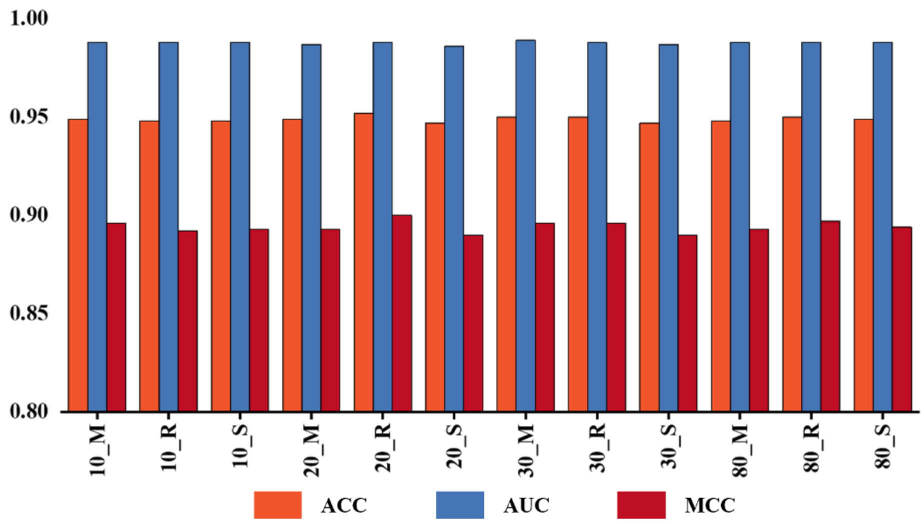

Figure S8. The evaluation of models based on three ways (fingerprint, random, scaffold) to select different percentages of added data.

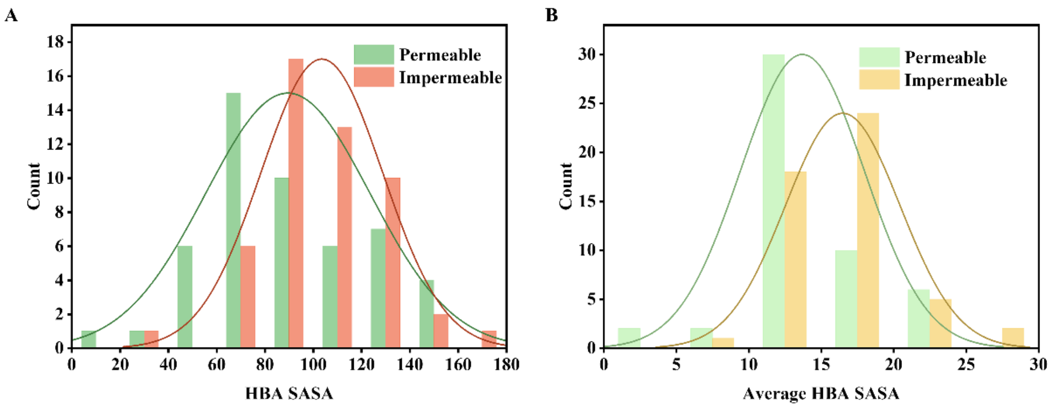

Figure S9. A, The distribution of HBA SASA. B, The distribution of average HBA SASA

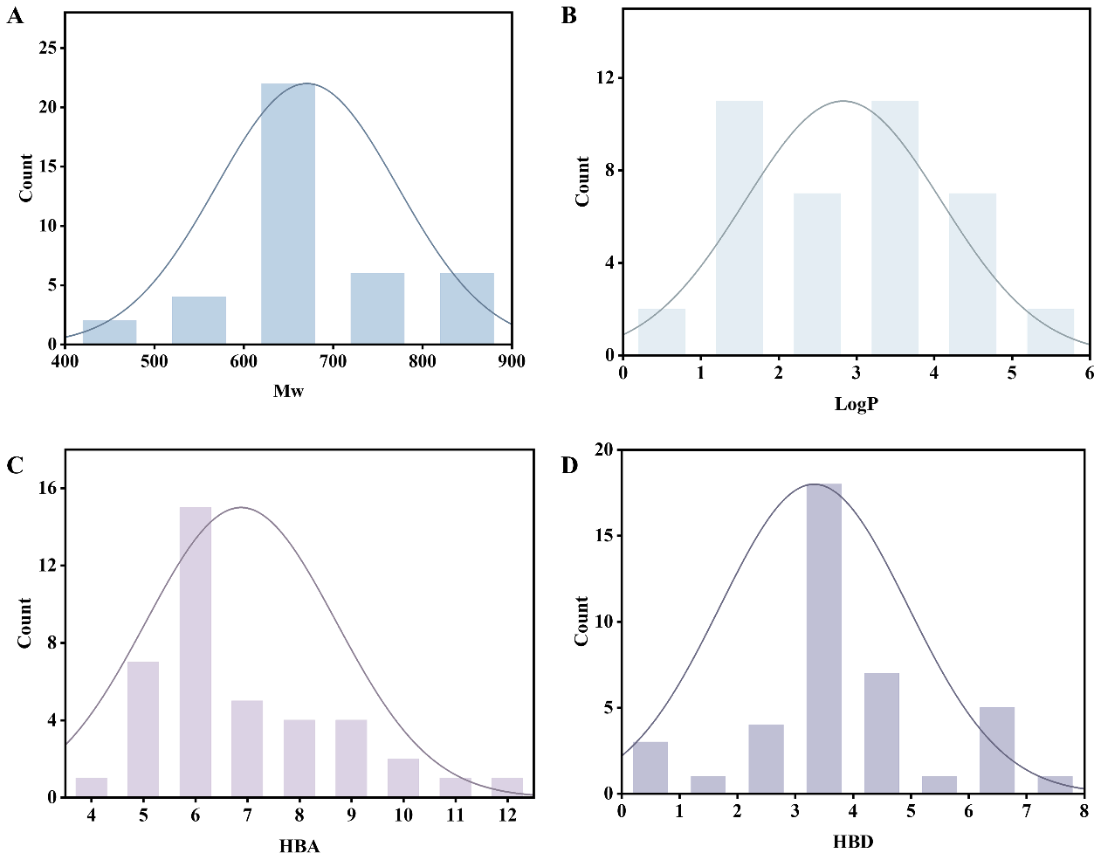

Figure S10. A, The distribution of HBA SASA. B, The distribution of average HBA SASA
